# The Antibacterial and Anti-Inflammatory Potential of *Cinnamomum camphora* chvar. *Borneol* Essential Oil In Vitro

**DOI:** 10.3390/plants14121880

**Published:** 2025-06-19

**Authors:** Shanshan Xiao, Hang Yu, Yahui Guo, Yuliang Cheng, Weirong Yao

**Affiliations:** 1 School of Food Science and Technology, Jiangnan University, No.1800 Lihu Avenue, Wuxi 214122, China; hangyu@jiangnan.edu.cn (H.Y.); guoyahui@jiangnan.edu.cn (Y.G.); ylcheng@jiangnan.edu.cn (Y.C.); 2 Faculty of Food Science and Engineering, Kunming University of Science and Technology, Kunming 650500, China; shine330@kust.edu.cn

**Keywords:** borneol, essential oil, *Staphylococcus epidermidis*, bacteriostatic activity, anti-inflammation, network pharmacology, primary/secondary factor analysis

## Abstract

*Cinnamomum camphora* chvar. *Borneol* essential oil (BEO, 16.4% borneol) is a by-product obtained during the steam distillation process used to produce natural crystalline borneol (NCB, 98.4% purity). This study aimed to compare the antibacterial activity of BEO and NCB against *Staphylococcus epidermidis*, and to evaluate the anti-inflammatory effect of BEO in vitro. Minimum inhibitory concentrations (MICs), determined by broth microdilution, were identical for both BEO and NCB (0.5 mg/mL). Despite this, BEO exhibited stronger antibacterial activity, suggesting synergistic enhancement by other components. Mechanistic studies revealed that BEO disrupted the bacterial cell wall, causing leakage of nucleic acids and proteins, and ultimately bacterial death. In LPS-induced RAW 264.7 macrophages, BEO dose-dependently reduced the production of TNF-α, IL-1β, and IL-6 (r = −0.9847, −0.9456, −0.9315). Network pharmacology, combined with primary and secondary factor analysis, was employed to identify anti-inflammatory pathways and key active compounds. Borneol contributed over 50% to the anti-inflammatory effect, followed by β-caryophyllene, limonene, camphor, and γ-terpinene. These findings highlight the potential enhanced bioactivity of BEO due to multi-component synergy.

## 1. Introduction

*Cinnamomum camphora* chvar. *Borneol* (*C. camphora*), a borneol-type chemotype of the camphor tree, is a medicinal plant native to East Asia and has been extensively used in traditional Chinese medicine (TCM) for centuries. Its branches and leaves are rich in natural borneol, a bicyclic monoterpene with diverse pharmacological activities. In TCM, borneol is regarded as a “guide drug” that facilitates the delivery of other medicinal compounds to target tissues, particularly the brain, due to its ability to cross the blood–brain barrier. Extracts from *C. camphora* exhibit a wide range of biological properties, including antimicrobial, anti-inflammatory, analgesic, and neuroprotective effects.

*Cinnamomum camphora* chvar. *borneol* essential oil (BEO, 16.4% borneol) is a by-product of steam distillation, which produces natural crystalline borneol (NCB, 98.4% borneol), and which has been shown to have significant anti-inflammatory effects [1]. The main active component is borneol (16.4%), followed by camphor (10.6%), β-caryophyllene (10.7%), limonene (8.2%), α-pinene (7.5%), and β-pinene (3.6%). NCB has broad-spectrum antibacterial activity, mediated primarily by disrupting the integrity of bacterial cell membranes [2], as well as strong anti-inflammatory activity in mice, by inhibiting carrageenan-induced migration of leukocytes into the peritoneal cavity [3].

Recent research has revealed that borneol can alleviate post-stroke neuroinflammation in mice by modulating immune cell polarization [4]. Similarly, β-caryophyllene has been shown to enhance the survival of random flaps by upregulating the PI3K/AKT signaling pathway, thereby improving tissue blood perfusion and reducing both inflammation and apoptosis [5]. Limonene has been widely reported to exhibit significant anti-inflammatory properties. For instance, a recent study demonstrated that limonene exerted anti-inflammatory effects by downregulating pro-inflammatory cytokines (TNF-α, IL-1β, and COX-2), inhibiting the TLR4/NF-κB/AP-1 signaling pathways, and modulating oxidative stress via Nrf2 activation [6]. In addition, α-pinene has been found to ameliorate liver fibrosis by suppressing oxidative stress and inflammation, as well as inhibiting the TGF-β/Smad3 signaling pathway [7].

Essential oils with similar composition to BEO have excellent antibacterial and anti-inflammatory properties and have long been used for the treatment of inflammation induced by bacterial infection [8]. Several essential oils have significant antibacterial activity and are considered safe and effective for the treatment of inflammatory conditions. The main focus of antibacterial research has been on *Staphylococcus epidermidis* (*S. epidermidis*), which, as the main causative agent of skin, soft tissue and systemic infections in humans, has become a major threat to human health, because of its resistance to several antibiotics [9]. *Salvia eremophila* essential oil, the major components of which are borneol (21.8%), α-pinene (18.8%), bornyl acetate (18.7%), camphene (6.5%), β-caryophyllene (4.5%) and limonene (2.7%) [4], and *Tanacetum argyrophyllum* var. *argyrophyllum* essential oil, the major components of which are borneol (15%), camphor (26.6%), and camphene (4.5%) [10], have significant antibacterial activity against *S. epidermidis*. Furthermore, 17% of clinical visits are primarily related to skin inflammation caused by bacterial infections and several essential oils are used for treatment, because of their combined anti-inflammatory and antibacterial effects [11]. *Origanum vulgare* L. essential oil, which has anti-*S. epidermidis* and anti-inflammatory properties, is used to treat acne [12]. Many plant essential oils are more effective, cheaper and have fewer side effects when used for the treatment of various diseases, compared with synthetic drugs [13]. BEO has a similar composition to the above-mentioned essential oils, so its antibacterial and anti-inflammatory activities are well worth exploring.

The main component of BEO (borneol, 16.4%) inhibits LPS-induced cellular inflammation and is associated with the cellular targets PTGS2, TRPA1 and TRPV1 [14]. The current recommended treatment for inflammation is combined antimicrobial and anti-inflammatory therapy, which not only reduces mutation of pathogenic bacteria and delays the development of resistance by acting on multiple therapeutic targets, but also reduces toxicity and adverse effects by lowering the required dose [11]. The “multi-component/multi-target/multi-signaling pathway” mechanism of action that characterizes essential oils could fulfill the requirement for novel antimicrobial and anti-inflammatory treatments for *S. epidermidis* skin infections.

This study aimed to investigate the therapeutic potential of BEO. The bacteriostatic differences between BEO and NCB were compared. Furthermore, the antibacterial properties and mechanisms of BEO, along with its effects on LPS-induced cellular inflammation, were evaluated. A component–target–signaling pathway network was constructed through network pharmacology to elucidate the material basis underlying BEO’s antibacterial and anti-inflammatory activities.

## 2. Results and Discussion

### 2.1. Chemical Composition of BEO

GC-MS/GC-FID analysis identified 42 components in BEO (Table S1), accounting for 96.73% of the total essential oil; the main components were borneol (16.4%), β-caryophyllene (10.7%), limonene (8.2%), α-pinene (7.5%), β-pinene (3.6%) and linalool (0.5%) (Table S1). These major components were also detected in the neutral cellulase-assisted steam distillation extract [15], with concentrations of borneol (11.7%), β-pinene (8.6%), and linalool (0.3%). The observed compositional variations may be attributed to differences in extraction methods, source materials, or seasonal factors.

### 2.2. Inhibitory Effect of BEO and NCB on S. epidermidis

#### 2.2.1. Minimum Inhibitory Concentration

The minimum inhibitory concentrations (MICs) of BEO and NCB against *S. epidermidis* were both 0.5 mg/mL (Table 1), indicating strong antibacterial activity. This value is comparable to the MICs of the commercial antibacterial agents rifampin and gentamicin against *S. epidermidis* [8], which are 0.25 and 0.5 mg/mL, respectively. Essential oils from *Tanacetum argyrophyllum* var. *argyrophyllum* [16] and *Salvia eremophila* [8], which have similar compositions to BEO, exhibit MICs of 0.5 and 0.125 mg/mL against *S. epidermidis*, respectively.

Analysis of the components shared by these essential oils revealed that borneol is the main active antibacterial constituent. Combined with a previous study on the antibacterial properties of NCB [2], it can be concluded that borneol is the primary antibacterial component. Camphene, β-pinene, spathulenol, and α-terpinene—common essential oil components present in similar proportions—appear to be the main components synergizing with borneol to exert antibacterial activity (Table 1). This synergistic interaction suggests that the potent antibacterial activity of BEO arises not only from borneol, but also from the cooperative effects of these minor constituents, which may contribute to reducing bacterial resistance and improving therapeutic potential.

#### 2.2.2. Inhibition of Microbial Growth

**Figure 1:**
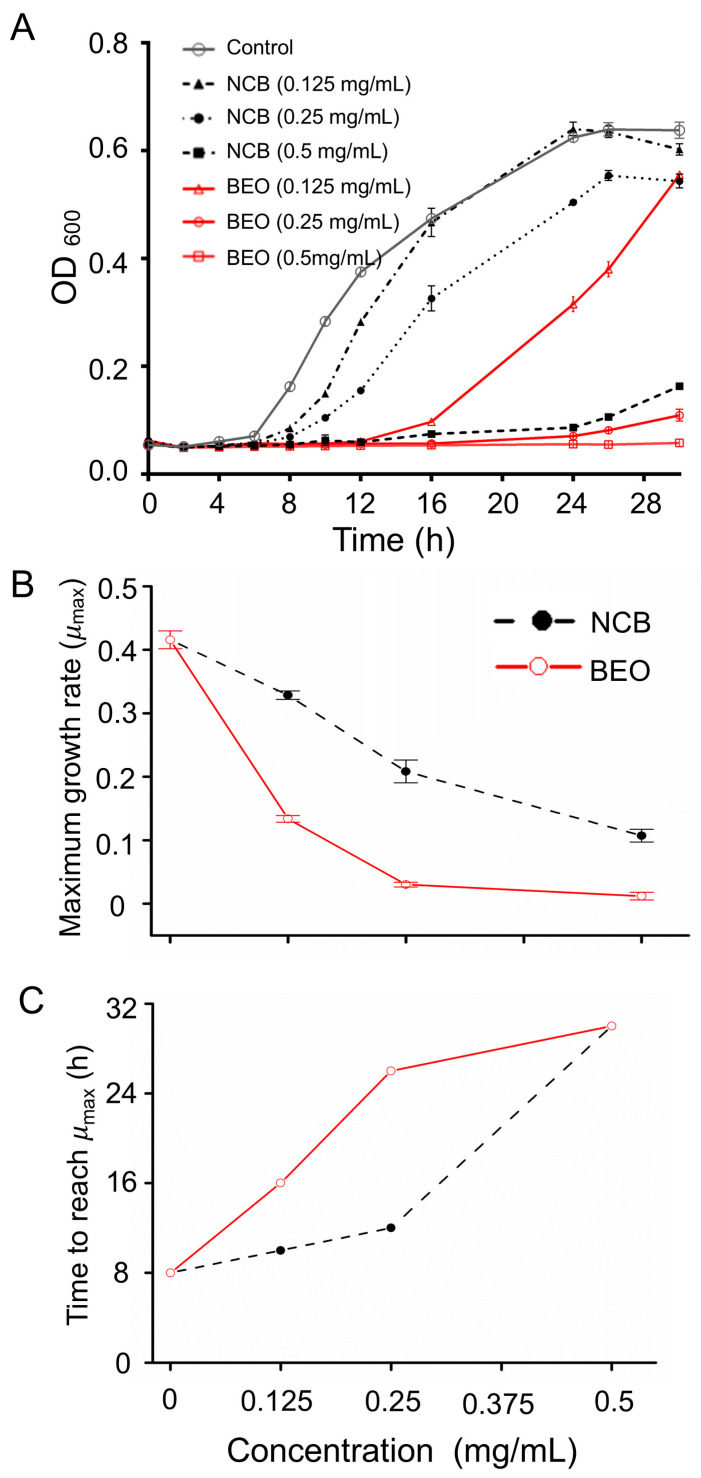
Effect of BEO and NCB on the cell growth of *S. epidermidis*. (**A**) Growth curves of *S. epidermidis* in the presence of different concentrations of BEO and NCB, determined from the optical density at 600 nm (OD_600_ nm). (**B**) Maximum growth rate (*μ*_max_) of *S. epidermidis*. (**C**) Culture time to reach the *μ*_max_ of *S. epidermidis*. Data are presented as mean ± standard error of the mean (SEM) from three independent experiments (*n* = 3).

The controls had an “S” type normal growth profile, entering the logarithmic phase after 8 h and the stationary phase after 24 h (Figure 1A). The growth of *S. epidermidis* treated with BEO and NCB at 0.125, 0.25 and 0.5 mg/mL was significantly inhibited, which prolonged the lag phase up to 30 h, with BEO at 0.25 and 0.5 mg/mL. Inhibition by BEO was significantly greater than by NCB at the same concentration, presumably as a result of a synergistic effect between the other components of the essential oil and borneol. The main components of essential oils that exhibit antibacterial activity are β-pinene, α-pinene, limonene, and γ-terpinene [17], all of which are present in BEO. In addition, the antibacterial activity of *L. formosana* oleoresin essential oil against *S. epidermidis* cannot be attributed to the activity of a single major component [18]. These results (Figure 1A) are in agreement with the MICs obtained previously.

The maximum growth rate (*μ*_max_) of *S. epidermidis* and the time to reach *μ*_max_ after NCB treatment were determined (Figure 1B). The *μ*_max_ of *S. epidermidis* in the control was 0.42 h^−1^ after 8 h of incubation, whereas the *μ*_max_ of 0.125, 0.25, and 0.5 mg/mL NCB-treated *S. epidermidis* was significantly reduced to 0.32 h^−1^, 0.21 h^−1^, and 0.11 h^−1^, respectively, after 10, 12, and 30 h of incubation (*p* < 0.0001). The corresponding parameters for BEO treatment were determined (Figure 1C). Compared with the controls, *μ*_max_ was significantly reduced to 0.13 h^−1^, 0.07 h^−1^ and 0.01 h^−1^, respectively, after 16, 26 and 30 h with 0.125, 0.25 and 0.5 mg/mL BEO treatment (*p* < 0.0001). Thus, after NCB and BEO treatment, the bacterial cell cycle was prolonged. The lower *μ*_max_ and more pronounced bacterial growth retardation after BEO treatment compared with NCB confirmed the superiority of BEO over NCB in terms of bacterial inhibition.

These results indicate that BEO exhibits superior antibacterial effects compared to NCB by prolonging the bacterial lag phase and significantly reducing the maximum growth rate. The synergistic action of multiple active components in BEO likely disrupts the integrity of the bacterial cell membrane and interferes with cellular energy metabolism and material transport, thereby inhibiting bacterial growth [19]. Furthermore, the growth curve analysis aligns with the MIC results, further confirming the potent antibacterial activity of BEO. This multi-component synergistic mechanism not only enhances antibacterial efficiency but also helps reduce the risk of developing resistant strains, thereby increasing its clinical potential as a natural antibacterial agent. It is noteworthy that this study used a standard strain; future work should extend to clinical isolates to assess the practical efficacy and safety of BEO in complex infection environments.

#### 2.2.3. Cell Membrane Integrity Determination by SYTO9/PI Staining/Laser Confocal Microscopy

**Figure 2:**
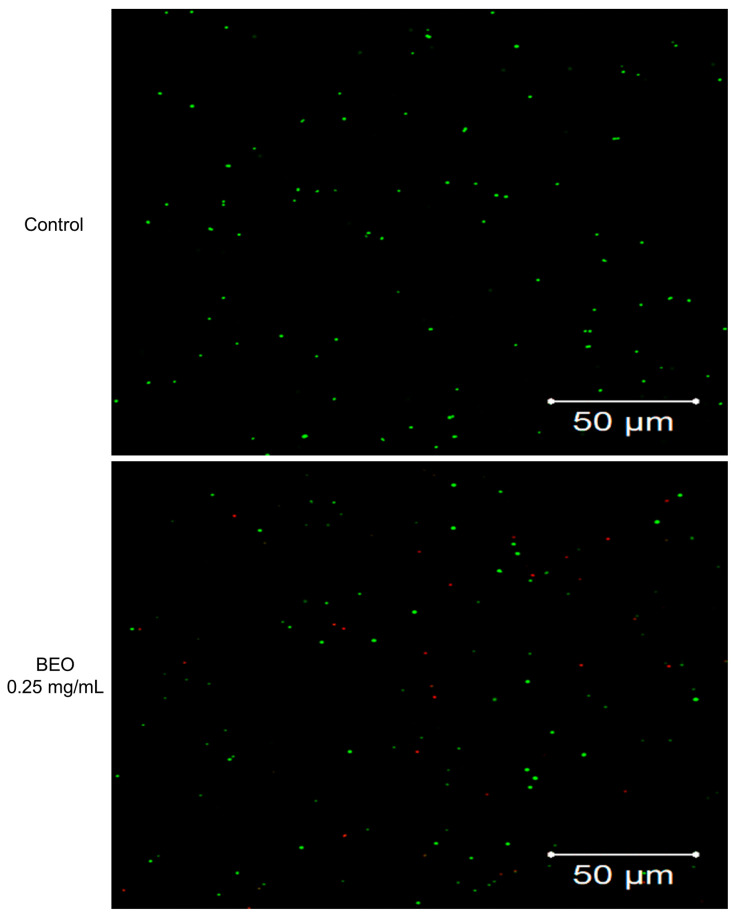
Bactericidal effects of BEO and NCB on *S. epidermidis* using CLSM and Live/Dead BacLight viability staining (SYTO9/PI). Bacteria with intact cell membranes are stained fluorescent green (SYTO9), whereas dead bacteria with damaged membranes are stained fluorescent red (PI). The scale bar is 50 μm.

SYTO9 and PI staining were used to determine the effect of BEO and NCB on *S. epidermidis* cell membrane integrity (Figure 2 and Figure S2); bacteria with intact cell membranes stain fluorescent green, whereas bacteria with damaged membranes stain fluorescent red [20]. Untreated control *S. epidermidis* cells only fluoresced green, but after treatment with BEO, or NCB, some cells fluoresced red, indicating cell death. As the concentrations of BEO and NCB increased, more red (dead) cells were observed and BEO treatment resulted in more dead cells than NCB treatment. These results were consistent with those from microbial growth measurements, indicating that BEO and NCB damage bacterial cell membranes and decrease viability.

The increased red fluorescence suggests that BEO exerts its bactericidal effect primarily by compromising the bacterial membrane, a mechanism commonly observed for essential oils. Membrane disruption not only leads to leakage of cellular contents but also impairs vital processes such as respiration and nutrient transport, ultimately leading to bacterial death [21].

The more pronounced membrane damage observed with BEO compared to NCB implies that synergistic interactions among its multiple constituents enhance its antibacterial efficacy. This multi-target mode of action is particularly advantageous, as it reduces the likelihood of bacteria developing resistance—a growing concern with conventional antibiotics [22]. Future mechanistic studies, such as lipid peroxidation analysis or membrane potential measurements, would provide deeper insights into the specific molecular events triggered by BEO.

### 2.3. Effect of BEO on Cell Membrane Integrity

#### 2.3.1. Leakage of UV-Absorbing Substances

**Figure 3:**
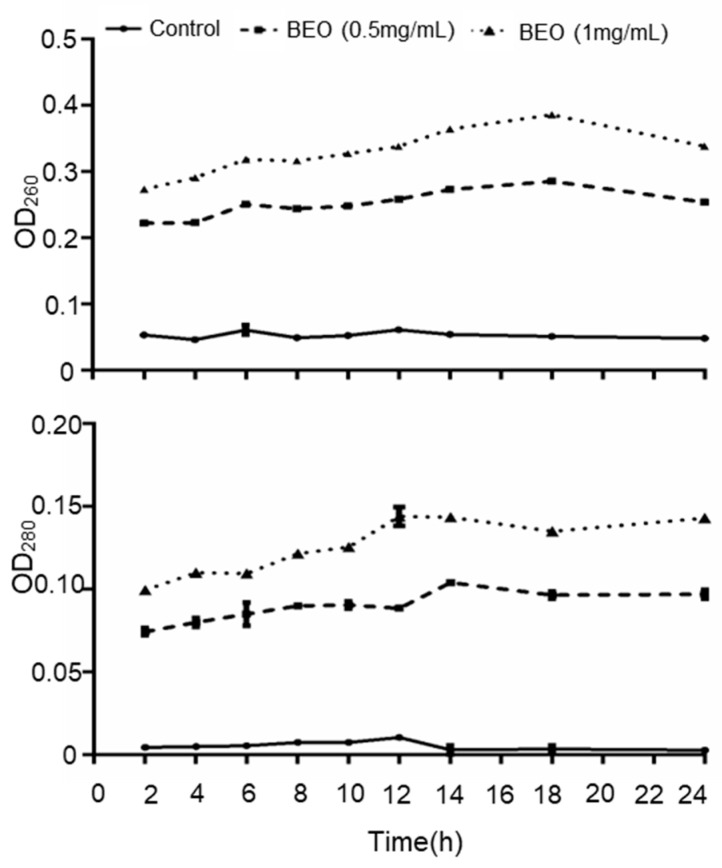
Effect of BEO on the leakage of intra-cellular UV-absorbing materials from *S. epidermidis*, detected at 260 nm (nucleic acid) and 280 nm (protein).

The cell membrane is an important structural component of bacteria. When the cell membrane is damaged, nucleic acids, proteins and other cytoplasmic contents leak out. Therefore, the leakage of intracellular substances is an important indicator of cell membrane integrity. Nucleic acids and protein have strong UV-absorption peaks at 260 and 280 nm, respectively, and measurement of these absorbances is a good indicator of cell membrane integrity [23]. In the untreated control, both absorbances were minimal and did not increase with time (Figure 3), whereas BEO treatment caused a marked increase in both absorbances, in a dose-dependent manner, indicating significant disruption of cell membrane integrity. *Cinnamon* essential oil [24] and *Cudrania tricuspidata* fruit essential oil [25] both exerted an antibacterial effect by disrupting cell membrane integrity, resulting in leakage of cytoplasmic nucleic acids and proteins.

These results further support the hypothesis that BEO’s antibacterial effect is primarily mediated through disruption of the bacterial cell membrane. Given the multi-component nature of essential oils, the membrane damage observed may result from synergistic interactions among lipophilic compounds that integrate into and destabilize the phospholipid bilayer. Future research employing techniques such as lipid peroxidation assays, membrane potential analysis, or transmission electron microscopy would help elucidate the precise molecular events underlying membrane disruption by BEO. These insights could contribute to the development of effective, natural antimicrobial agents suitable for use in pharmaceutical or food preservation applications.

#### 2.3.2. Observation of BEO-Induced Cell Damage by Scanning Electron Microscopy (SEM) and Transmission Electron Microscopy (TEM)

**Figure 4:**
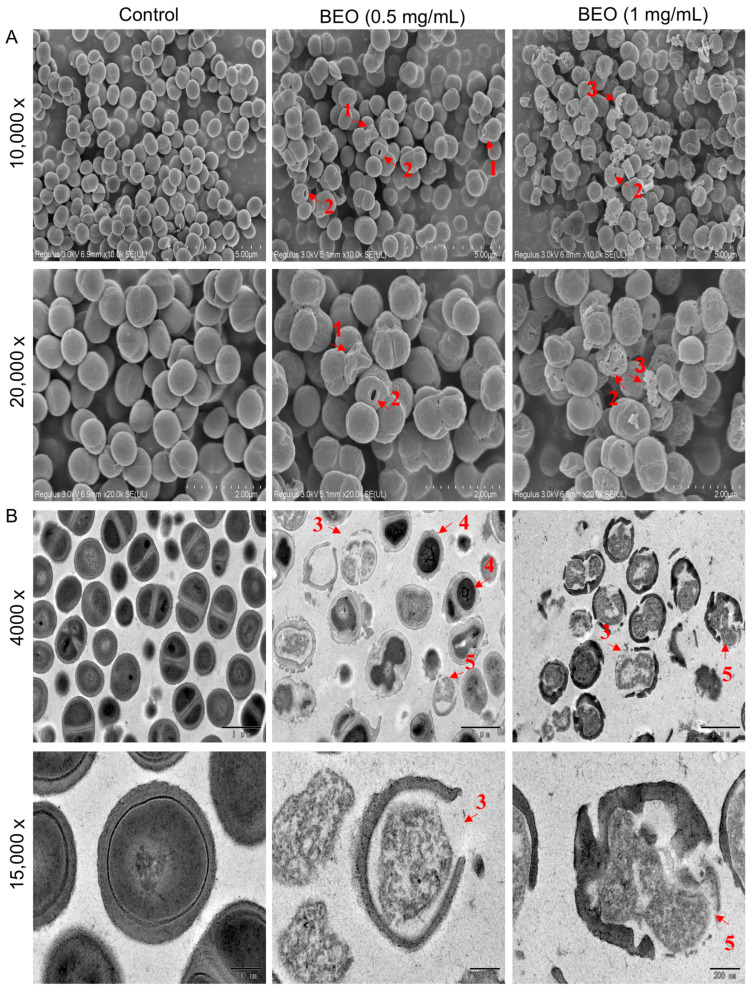
Scanning electron micrographs (**A**) and transmission electron micrographs (**B**) of untreated *S. epidermidis* cells (Control) and cells treated with BEO (0.5 mg/mL) and (1 mg/mL). Wrinkled cell wall (arrows #1), holes (arrows #2), discontinuity, ruptured appearance on the cell surface (arrows #3), condensation of nuclear material (arrows #4) and loss of cytoplasm (arrows #5) are visible.

SEM and TEM observations were performed on the cells treated with BEO (Figure 4). The untreated control cells maintained a normal spherical structure with a smooth surface, whereas after BEO treatment at 0.5 and 1 mg/mL, the cell surface became rougher and some cells were shriveled, indicating perforation and collapse of the cell wall (Figure 4A, red arrows #1). Holes were visible on some cells (Figure 4A, red arrows #2). After BEO treatment at 1 mg/mL, some cells were ruptured and fragments of cell wall were visible (Figure 4A, red arrow #3). It appears that BEO caused various degrees of cell membrane damage ranging from minor surface irregularities to complete destruction. Similarly, the inhibitory effect of *bifidocin* A on *E. coli* appears to be related to the formation of pores in the cytoplasmic membrane [20]. In addition, the active components of essential oils appear to act by binding to the cell surface, then penetrating to target sites on the plasma membrane and membrane-bound enzymes, resulting in disruption of the cell structure [25].

In the TEM images (Figure 4B) of the control, the cytoplasm appeared evenly distributed and the cell walls were intact. After BEO treatment at 0.5 and 1mg/mL, condensation of nuclear material and disruption of the cell wall (Figure 4B, red arrows #3), loss of cytoplasm (Figure 4B, red arrows #4), and bulging of cell membranes through breaks in the cell wall (Figure 4B, red arrows #5) were observed, suggesting the failure of cell and nuclear division. This is consistent with previous findings on the inhibitory effect of *bifidocin* A on *E. coli* 16 and with the above finding of leakage of cytoplasm contents after BEO treatment.

### 2.4. Effect of BEO on LPS-Induced Inflammation in RAW 264.7 Mouse Macrophage Cells

#### 2.4.1. Effect of BEO on RAW 264.7 Cell Viability

**Figure 5:**
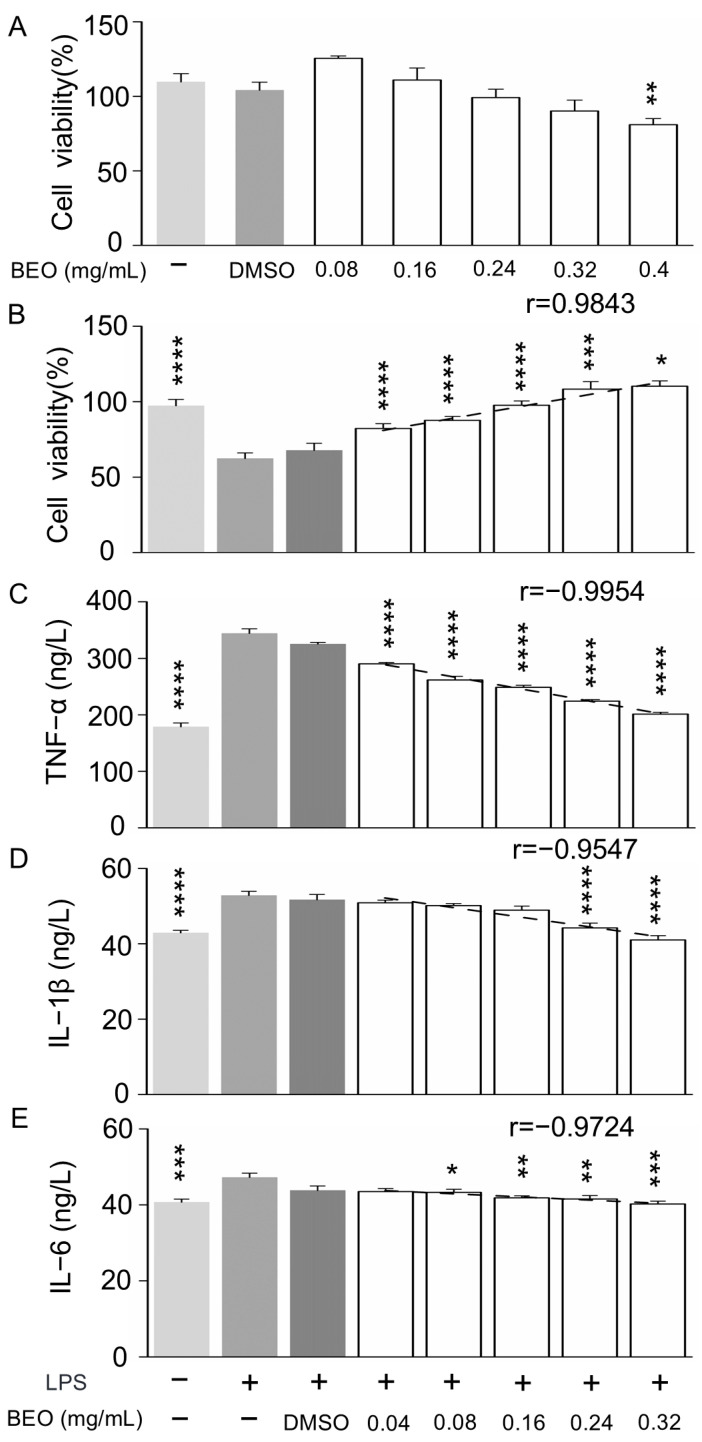
BEO alleviated LPS-induced cell injury in RAW 264.7 cells. (**A**) Viability of BEO-treated RAW 264.7 cells, determined by the Cell Counting Kit-8 assay. (**B**) Viability of LPS-induced RAW 264.7 cells after BEO treatment, determined by the Cell Counting Kit-8 assay. The concentrations of tumor necrosis factor α (TNF-α) (**C**), interleukin-Iβ (IL-1β) (**D**) and IL-6 (**E**), determined by ELISA. Data are expressed as the mean ± SEM. *n* = 3. Statistical analysis was done by one-way analysis of variance with Dunnett’s multiple comparisons test, * *p* < 0.05, ** *p* < 0.01, *** *p* < 0.001, **** *p* < 0.0001 compared to LPS alone.

According to Kuete and Efferth (2015) [26], plant extracts with half maximal inhibitory concentration (IC_50_) values higher than 400 μg/mL are considered non-toxic. At a BEO concentration of 400 μg/mL, cell viability decreased to 80.94% ± 0.04% (*p* < 0.01) of the control value (Figure 5A). In contrast, the survival rates of RAW 264.7 cells at the BEO concentrations of 0.08, 0.16, 0.24 and 0.32 mg/mL were not significantly different from the control (*p* > 0.05). Therefore, it can be concluded that BEO is safe at concentrations below 0.4 mg/mL and that 0.32 mg/mL BEO, or less, was suitable for subsequent studies of cellular inflammation.

These findings indicate that BEO exhibits low cytotoxicity at the tested concentrations, which is critical for its potential therapeutic application. Maintaining high cell viability ensures that observed anti-inflammatory effects are not masked by cytotoxicity. This is especially important in macrophage models, where cell health directly influences inflammatory signaling pathways. Moreover, the safety profile of BEO supports its development as a natural anti-inflammatory agent, which aligns with previous studies demonstrating the low toxicity of essential oils at appropriate doses [27]. Future studies should also investigate BEO’s effects on primary macrophages and in vivo models to further validate its safety and efficacy.

#### 2.4.2. Effect of BEO on LPS-Induced Inflammation in RAW 264.7 Cells

The effect of LPS-stimulation on cell viability was determined by Cell Counting Kit-8 measurements (Figure 5B). Viability significantly decreased in the LPS model cells, compared with control cells, which is consistent with a previous study [28]. BEO treatment significantly inhibited the LPS-induced decrease in cell viability, in a dose-dependent manner (r = 0.9843), indicating that BEO has a protective effect against cellular inflammatory damage caused by LPS.

Determination of inflammatory mediators in cell supernatants showed that LPS significantly increased the release of TNF-α (Figure 5C), IL-1β (Figure 5D) and IL-6 (Figure 5E) compared with the control (*p* < 0.05), whereas BEO treatment inhibited the LPS-induced secretion of excess inflammatory factors in a dose-dependent manner (r = −0.9954, r = −0.9547, r = −0.9724, respectively). BEO at 0.04, 0.08, 0.16, 0.24, 0.32 mg/mL significantly inhibited the secretion of TNF-α, by 15.7 ± 0.02, 23.9 ± 0.04, 27.7 ± 0.01, 34.8 ± 0.01 and 41.5 ± 0.01%, respectively. BEO at 0.24 and 0.32 mg/mL significantly inhibited the secretion of IL-1β, by 16.3 ± 0.03 and 22.8 ± 0.01%, respectively. BEO at 0.08, 0.16, 0.24 and 0.32 mg/mL significantly inhibited the secretion of IL-6, by 8 ± 0.04, 8.3 ± 0.01, 11.4 ± 0.04 and 14.9 ± 0.01%, respectively, indicating that BEO inhibited the LPS-induced inflammatory response in RAW 264.7 cells.

In a previous study of borneol inhibition of LPS-induced inflammation in RAW 264.7 cells, 25, 50 and 100 μg/mL of borneol significantly inhibited the LPS-induced release of TNF-α (by 11–22%), IL-1β (by 10–36%) and IL-6 (by 15–23%) [29], similar to the results from this study. The concentrations of borneol in the BEO treatments used here were 6, 13, 26, 39, and 52 μg/mL, so BEO appears to have stronger anti-inflammatory activity than borneol, suggesting synergistic effects with other components. Moreover, these results are consistent with the effects on the microbial growth curve (Figure 1).

### 2.5. Network Pharmacology Analysis

Network pharmacology is a practical approach to explore the therapeutic mechanisms of multi-component traditional Chinese medicines in the treatment of complex diseases. We used network pharmacology analysis to predict the potential targets and pathways of action for the anti-inflammatory effects of BEO.

#### 2.5.1. BEO Constituents and Their Therapeutic Targets

**Figure 6:**
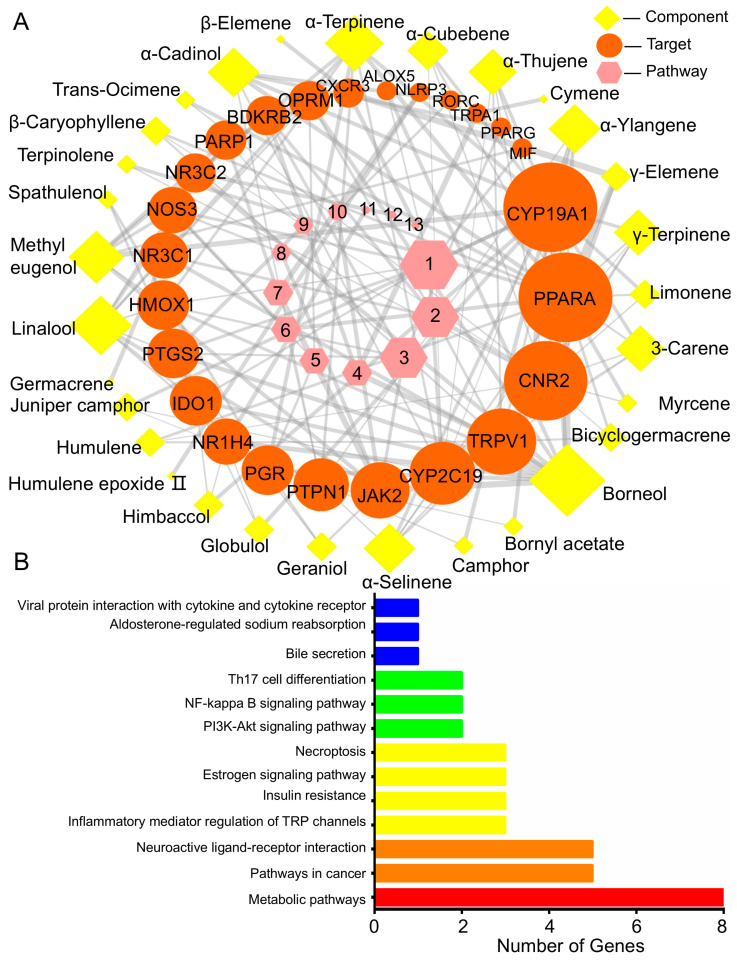
Inflammation pharmacology network of the “compounds–targets–pathways” regulated by BEO. (**A**) Anti-inflammatory network of the “components-targets-signaling pathways” regu-lated by BEO. Yellow rhomboidal nodes represent compounds, orange round nodes represent targets, and pink hexagonal nodes represent pathways. 1. Metabolic pathways; 2. Pathways in cancer; 3. Neuroactive ligand-receptor interaction; 4. Inflammatory mediator regulation of TRP channels; 5. Insulin resistance; 6. Estrogen signaling pathway; 7. Necroptosis; 8. PI3K-Akt signaling pathway; 9. NF-kappa B signaling pathway; 10. Th17 cell differentiation; 11. Bile secretion; 12. Aldosterone-regulated sodium reabsorption; 13. Viral protein interaction with cytokine and cytokine receptor. (**B**) The top 13 KEGG pathways associated with the enrichment of the therapeutic targets.

In this study, based on the GC-MS compositional analysis of BEO (Table S1), the potential active components of BEO were screened from the database and an “active ingredient–target–signaling pathway” network was constructed (Figure 6A, Table S2). A total of 30 BEO anti-inflammatory components and 25 corresponding targets of action were obtained. Among the 30 active components of BEO, the most abundant component, borneol (16.4%), was linked to the largest number of targets (13) and can therefore be considered as the main anti-inflammatory component.

The target genes Cytochrome P450 (CYP)19A1 and Peroxisome proliferator-activated receptor-α (PPARA) were associated with 17 BEO components; cannabinoid receptor 2 (CNR2) was associated with 14 BEO components; transient receptor potential cation channel (TRP)V1 and CYP2C19 were associated with nine BEO components. Diet-induced obesity and inflammation increase CYP19A1 gene expression in subcutaneous and abdominal white fat in men, showing a close link between CYP19A1 and inflammation. PPAR-α is a member of the nuclear hormone receptor superfamily, which is involved in the regulation of lipid metabolism, adipocyte differentiation and inflammatory processes, through the activation of related transcription factors. CNR2 is a potential target for the development of therapeutic agents for periodontitis and other oral inflammatory diseases [30]. TRPV1 is a non-selective cation channel, mainly located in sensory nerve endings which, upon activation, releases a variety of neurotransmitters in atherosclerosis; TRPV1 activation regulates lipid metabolism, reduces foam cell formation, protects endothelial cells, inhibits smooth muscle cell proliferation and suppresses the inflammatory response. In addition, competitive binding between esters synthesized from borneol and glycine and TRPA1/TRPV1 agonists is responsible for their significant analgesic effect [31]. Limonene and methyl eugenol are TRPA1 agonists [32].

The above reports support the reliability of the network pharmacological analysis, by validating the links between the identified targets and inflammation. In addition, the relationships between BEO components and their therapeutic targets reflect its multi-component and multi-target action.

#### 2.5.2. Target- Pathway Cascade Analysis in Network Pharmacology

KEGG pathway analysis (Figure 6B) showed that the 25 therapeutic targets identified by network pharmacological analysis were mainly associated with the following pathways: metabolic pathways (eight targets); pathways in cancer (five targets); neuroactive ligand-receptor interaction (five targets); inflammatory mediator regulation of TRP channels (three targets); insulin resistance (three targets); estrogen signaling pathway (three targets); necroptosis (three targets); PI3K-Akt signaling pathway (two targets); NF-kappa B signaling pathway (two targets); Th17 cell differentiation (two targets). Clearly, the targets of BEO active components are distributed over many different pathways and act in a coordinated manner. This pathway analysis is consistent with previous reports associating pathways with inflammation, i.e., metabolic pathways are considered as potential targets to regulate the immune system by either enhancing or suppressing the immune response, and many cancers originate from sites of inflammation, chronic irritation, or infection [33].

Another important interaction is that between the immune system and the nervous system. In addition to immune cells, neural tissues synthesize neuropeptides and cytokines, which are the molecular basis of the interaction between the nervous and immune systems. Neuromodulation has both pro-inflammatory and anti-inflammatory effects [34]. Inflammatory mediator regulation of TRP channels is mainly associated with nociceptive hypersensitivity, inflammation and cirrhosis [31]. Inflammation leads to insulin resistance and metabolic disorders, which are associated with changes in the immune cells found in adipose tissue, and this inflammation in adipose tissue is probably triggered by NLRP3 inflammasome activation.

The estrogen signaling pathway regulates many physiological processes, such as reproduction, cardiovascular protection, cellular homeostasis and inflammatory metabolic processes. Necroptosis is a type of programmed cell death associated with inflammation [35]. The PI3K-Akt and NF-κB signaling pathways and Th17 cell differentiation are involved in immune and inflammatory responses [36,37]. This analysis of the relevant pathways is largely consistent with previous reports and provides new insights into the potential anti-inflammatory mechanisms of BEO.

The “component–target–signaling pathways” related to the regulation of inflammation by NCB, *Tanacetum argyrophyllum* var. *argyrophyllum* essential oil and *Salvia eremophila* essential oil, were constructed by network pharmacology, and the material basis of their anti-inflammatory effects was analyzed. Two components in NCB acted on thirteen targets (Figure S1 and Table S3); twenty-one components in *T. argyrophyllum* essential oil acted on twenty-eight targets (Figure S3 and Table S4). Nineteen components in *Salvia eremophila* essential oil acted on twenty-six targets (Figure S4 and Table S5) and its anti-inflammatory targets and pathways were very similar to those of BEO (Table S2). Based on this analysis, the material basis of BEO’s anti-inflammatory effect was further elucidated.

According to the Activity Based Classification (primary and secondary factor analysis method, 80% of which is considered to be the main influencing factor), a weight analysis of the BEO anti-inflammatory active components was carried out. Of the main active components screened (Table S6), the five highest contributors were borneol (component target weight (CTW) 55.83%, component pathway weight (CPW) 55.1%); β-caryophyllene (CTW 8.53%, CPW 8.99%); limonene (CTW 6.54%, CPW 6.89%); camphor (CTW 5.53%, CPW 5.94%); γ-terpinene (CTW 4.88%, CPW 4.14%).

Using the same screening method (Table S6), the main anti-inflammatory component in NCB was found to be borneol (CTW 99.88%, CPW 99.86%). There were two main anti-inflammatory components in *T. argyrophyllum* essential oil, borneol (CTW 66.17%, CPW 67.9%) and camphor (CTW 17.68%, CPW 18.52%). *Salvia eremophila* essential oil had four main anti-inflammatory components, borneol (CTW 68.28%, CPW 65.59%), bornyl acetate (CTW 8.87%, CPW 10.23%), linalool (CTW 3.25%, CPW 3.69%) and β-caryophyllene (CTW 3.25%, CPW 3.69%).

An anti-inflammatory “component-target–signaling pathway” network for BEO was constructed by network pharmacology analysis and the contribution of borneol to the anti-inflammatory effect was found to be the highest (more than 50%) when comparing BEO with three other antibacterial essential oils containing similar components, both in terms of component target weight and component pathways weight; other components (β-caryophyllene, limonene, camphor and γ-terpinene) also contributed. The analysis also revealed the main targets of BEO action to be CYP19A1 and PPAR-α, and the main pathways of action were metabolic pathways. These findings provide greater understanding of the mechanism of action and material basis of BEO’s anti-inflammatory activity.

## 3. Materials and Methods

### 3.1. Materials and Reagents

*C. camphora* trees were planted in the Shaoxing Plant Base (Zhejiang, China, 29.9° N, 120.8° E). BEO and NCB were produced by Chunjingziran Biotechnology Co., Ltd. (Zhejiang, China) via industrial steam distillation of fresh branches and leaves (Figure S5) harvested in October 2021. During the distillation process, natural borneol was collected as the primary product, while BEO was obtained as a by-product through condensation of volatile components. The essential oil layer was separated, dried over anhydrous sodium sulfate to remove residual moisture, and stored in airtight amber glass bottles at 4 °C until use. No further chemical modification or purification was performed prior to analysis. The yield of BEO was approximately 0.65% [15]. A voucher specimen (No. 768133) of *C. camphora* has been deposited at the South China Institute of Botany, Chinese Academy of Sciences (Guangzhou, China).

Reagents used were Dulbecco’s Modified Eagle’s Medium (DMEM; GIBCO/Thermo Fisher Scientific, Waltham, MA, USA); fetal bovine serum (FBS; GIBCO); penicillin/streptomycin (GIBCO); dimethyl sulfoxide (DMSO; Solarbio, Beijing, China); Cell Counting Kit-8 (Beyotime, Shanghai, China); lipopolysaccharide (LPS; *Escherichia coli* 055:B5, Solarbio, Beijing, China) and enzyme-linked immunosorbent assay (ELISA) kits (SenBeiJia Biotechnology, Nanjing, China), which were used to detect tumor necrosis factor (TNF)-α, interleukin (IL)-1β and IL-6.

### 3.2. Chemical Compositional Analysis of BEO

Gas chromatography/mass spectrometry (GC-MS) was carried out using an Agilent 7890B gas chromatograph (Agilent Technologies, Santa Clara, CA, USA) equipped with a DB-Wax fused-silica capillary column (30 m × 0.25 mm × 0.25 μm) that was directly connected to a time-of-flight mass spectrometer (Pegasus BT, LECO Instruments, Geleen, The Netherlands). The oven temperature program was as follows: initial temperature 45 °C (0–2 min), increasing to 230 °C at 8 °C/min, held for 10 min; carrier gas (He) flow rate 1.0 mL/min, inlet temperature 250 °C. The extraction head was desorbed at 250 °C for 3 min before injection and the undiluted liquid sample (0.5 μL) was directly injected. MS conditions were as follows: Electron impact (EI) ionization, interface temperature 250 °C, ion source temperature 200 °C, emission current 100 μA, electron energy 70 eV, detector voltage 1000 V, scanning mass range 33–450 amu [1].

GC-FID analysis was accomplished on a Shimadzu (Kyoto, Japan) GC2010, equipped with a flame ionization detector (FID). The detector temperature was 230 °C. The other analytical conditions, including column type and temperature, injector temperature, carrier gas and flow rate, were the same as for the GC–MS analysis.

The BEO components were divided into four categories (monoterpenes, oxygenated monoterpenes, sesquiterpenes, and oxygenated sesquiterpenes). A mixture of n-alkanes (C6–C26) was analyzed under the same conditions, to calculate the retention index (RI) for each peak. RIs were documented in the National Institute of Standards and Technology (NIST) WebBook Database (https://webbook.nist.gov/chemistry/ (accessed on 1 March 2021)) [38].

### 3.3. Bacterial Strains and Culturing

The standard strain of *S. epidermidis* (ATCC 12228) used in this study was obtained from the Guangdong microbial culture collection center. The strain was cultured aerobically on nutrient broth (NB) medium and incubated at 37 °C for 24 h.

### 3.4. Antibacterial Effect of BEO on S. epidermidis

#### 3.4.1. Determination of the MIC of BEO and NCB

The minimum inhibitory concentration (MIC) of BEO and NCB was determined by the two-fold serial dilution method [25]. BEO and NCB were first dissolved in DMSO and incorporated into NB medium for bacterial pathogens to obtain a concentration of 8 mg/mL, then serially diluted to achieve 4, 2, 1, 0.5, 0.25 and 0.125 mg/mL, respectively. A 100 μL standardized suspension of *S. epidermidis* (approximately 10^6^ colony forming units (CFU)/mL) was transferred to each well. The bacterial suspensions were incubated at 37 °C for 24 h. The lowest concentration at which no visible growth of *S. epidermidis* could be observed was designated as its MIC, expressed in mg/mL.

#### 3.4.2. Measurement of the Growth Curve

The inhibitory effect of BEO and NCB on the growth of *S. epidermidis* was evaluated using a spectrophotometric method, as described previously [32,33], with minor modifications. Briefly, logarithmic phase *S. epidermidis* was harvested by centrifugation (8000× *g*, 4 °C, 5 min) and adjusted to 10^8^ CFU/mL with normal saline. Bacterial suspension (2 mL) was inoculated into flasks containing fresh sterile nutrient broth (200 mL). BEO and NCB were added to the culture to achieve final concentrations of 0.125, 0.25 and 0.5 mg/mL. An *S. epidermidis* culture with 0.5% DMSO was used as the control. All cultures were incubated on an orbital shaker (120 rpm at 37 °C). The growth rates and the bacterial concentrations were monitored every 2 h by measuring the absorbance at 600 nm (OD_600_) using a UV/visible spectrophotometer (UV-1800, Shimadzu, Kyoto, Japan). The growth rate (*μ*) of *S. epidermidis* in the presence of various concentrations of BEO and NCB was calculated using OD_600_ values according to the following equation [39,40]:

μ = (lnA_1_ − lnA_2_)/(t_1_ − t_2_)

where A_1_ and A_2_ are the OD_600_ nm values at culture times t_1_ and t_2_, respectively.

#### 3.4.3. Effect of BEO and NCB Treatment on Cell Membrane Integrity

To assess the damage to *S. epidermidis* cell membranes following treatment with BEO and NCB, confocal laser scanning microscopy (CLSM) analyses were performed using a LIVE/DEAD BacLightTM Bacterial Viability Kit (L7012, Thermo) according to the manufacturer’s protocol [26]. Cultured *S. epidermidis* cells were harvested by centrifugation (as above), washed with 0.85% saline, then a mixture of SYTO9 and propidium iodide (PI) was added, followed by incubation at room temperature for 20–25 min in the dark. The mixture of SYTO9 and propidium iodide stains bacteria with intact cell membranes fluorescent green, whereas bacteria with damaged membranes are stained fluorescent red. The excitation/emission maxima for these dyes are 488/528 nm for SYTO9 stain and 488/645 nm for PI. Fluorescence measurements were made with a Zeiss (Jena, Germany) LSM 880 CSLM.

#### 3.4.4. Determination of Bacterial Cell Membrane Integrity

The integrity of the bacterial cell membrane was determined by ultraviolet absorption, as described previously [26], with minor modifications. Briefly, logarithmic phase *S. epidermidis* cells were harvested by centrifugation (as above) and adjusted to 107 CFU/mL with culture medium. BEO was dissolved in DMSO and added to the culture medium to achieve final concentrations of 0.5 and 1 mg/mL, respectively, and medium containing only 0.5% DMSO was used as the control. After incubation for 24 h at 37 °C with constant shaking (120 rpm), the culture medium supernatant was collected (8000× *g*, 4 °C, 5 min) by centrifugation every 2 h and the absorbance measured at 260 and 280 nm, with a UV/visible spectrophotometer (UV-1800, Shimadzu, Japan), to quantify nucleic acids and protein that had leaked from the cells. The higher the absorbance, the more severe the cell membrane damage.

#### 3.4.5. Scanning Electron Microscopy (SEM)

SEM was performed as described previously [24], with minor modifications. Briefly, logarithmic phase *S. epidermidis* was harvested by centrifugation (as above) and adjusted to 10^7^ CFU/mL with culture medium. BEO was dissolved in DMSO and added to the culture medium to achieve final concentrations of 0.5 and 1 mg/mL, respectively, and medium containing only 0.5% DMSO was used as the control. After incubation for 24 h at 37 °C with constant shaking (120 rpm), samples (1.5 mL) were centrifuged (as above) and the bacterial cell pellet resuspended with 2.5% glutaraldehyde solution, fixed at 4 °C for 10 h, then washed three times with phosphate buffer (pH 7.4). The cells were dehydrated by washing successively with 30, 50, 70, 90 and 100% aqueous ethanol, lyophilized, coated with gold and examined with a scanning electron microscope (Hitachi S-4800 FESEM, HITACHI, Tokyo, Japan), to observe the morphology of the bacteria.

#### 3.4.6. Transmission Electron Microscopy (TEM)

TEM was used to observe changes in the intracellular organization of *S. epidermidis* cells treated with BEO, as described previously [20], with some modifications. Control and BEO-treated cells were harvested and processed as described (Section 3.4.5) up to the glutaraldehyde fixing stage, then fixed with osmic acid, dehydrated, soaked, embedded and ultrathin sections prepared. The samples were processed by the negative staining method, then observed with a JEM-1200EX transmission electron microscope (Japan Electronics Co., Ltd., Tokyo, Japan).

### 3.5. Effect of BEO In Vitro on LPS-Induced Inflammation in Murine Macrophages (RAW 264.7)

#### 3.5.1. Cell Culture

The RAW 264.7 murine macrophage cell line was provided by the Kunming Institute of Zoology, Chinese Academy of Sciences (Kunming, China). RAW 264.7 cells were cultured in DMEM supplemented with 10% FBS and penicillin/streptomycin at 37 °C in a humid 5% CO_2_ atmosphere.

#### 3.5.2. Cell Viability Assay After LPS Treatment

The effects of BEO on the viability of RAW 264.7 cells were analyzed with Cell Counting Kit-8. RAW 264.7 cells were seeded in 96-well plates at 5000 cells/well. BEO solubilized in DMSO (<0.1% of total volume of cell suspension) was diluted in DMEM prior to treatment. BEO (0, 0.04, 0.08, 0.16, 0.24 or 0.32 mg/mL) was added to pretreat the cells and incubated for 1 h. Treated cells were stimulated with LPS (1 μg/mL), control cells were not. After 24 h, the absorbance was measured using a microplate reader (Epoch 2, Bio Tek Instruments, Winooski, VT, USA) at 450 nm. Concentrations were determined for three wells of each sample and each experiment was performed in triplicate.

#### 3.5.3. Cytokine Measurement in RAW 264.7 Cells After LPS Treatment

RAW 264.7 cells were seeded into a 96-well plate at a density of 5000 cells/well and incubated overnight prior to treatment. Cells were treated with 0, 0.04, 0.08, 0.16, 0.24 or 0.32 mg/mL BEO and 1 μg/mL LPS for 24 h. The supernatant was transferred to an ELISA plate and TNF-α, IL-Iβ and IL-6 in the culture medium were determined using an ELISA kit according to the manufacturer’s instructions.

### 3.6. Network Pharmacology Analysis

Simplified Molecular Input Line Entry System (SMILES) strings of each component were obtained by searching the traditional Chinese medicine Integrated Database (https://tcmbank.cn// (accessed on 1 March 2021)) and imported into the Swiss Target Prediction database (STP; http://www.swisstargetprediction.ch/ (accessed on 1 March 2021)) to identify potential targets for BEO components. The STP database can predict the targets of active molecules based on their chemical structure, ligand similarity and by cross-validation and arrangement analysis [41]. The predicted targets of all BEO components were obtained from limited search species in humans. Next, the DisGeNET database (http://www.disgenet.org/web/DisGeNET/menu/home (accessed on 1 March 2021)) was used to screen potential targets for inflammation, after which the targets of BEO components and of inflammation were intersected to identify potential inflammation targets that could be treated by anti-inflammatory BEO components.

The KEGG Mapper tool of the KEGG database (http://www.kegg.jp/ (accessed on 1 March 2021)) was used to find enriched pathways for the identified targets and Cytoscape 3.2.1 was then used to construct an “active components–targets–signal pathways” network, in which nodes representing BEO active components, potential targets and associated signal pathways were connected [42]. Potential mechanisms of BEO components for anti-inflammatory effects were determined by analyzing the resulting network. The contribution weights were calculated according to the following formulae.

Normalized value of the number of targets for each component:

Nti = Nt1 × 100/∑Nt1 − n

Normalized value of the number of pathways for each component:

Npi = Np 1 × 100/∑Np 1 − n

Contribution weight of each component to the number of targets:

Ti = Nti × Ci/∑(Nt1 × C1, …, Ntn × Cn) × 100

Contribution weight of the number of pathways for each component:

Pi = Npi × Ci/∑(Np1 × C1, …, Npn × Cn) × 100

where Nt is the number of target sites corresponding to each component; Np is the number of pathways corresponding to each component; C is the relative percentage of each component (%).

### 3.7. Data Analysis

Prism 6 software (GraphPad, San Diego, CA, USA) and OriginLab-9.0s (OriginLab, Northampton, MA, USA) were used for data analysis and plotting. Results are expressed as the mean ± standard deviation. The data were analyzed by using one-way analysis of variance with Dunnett’s multiple comparisons test. Pearson correlation analysis was performed with SPSS (Version 17.0, SPSS Inc., Chicago, IL, USA). * *p* < 0.05; ** *p* < 0.01; *** *p* < 0.001; **** *p* < 0.0001 were considered as statistically significant.

## 4. Conclusions

This study highlights the potential of BEO as a promising candidate for developing safe and effective topical formulations with dual antibacterial and anti-inflammatory functions. By demonstrating its inhibitory effects on *S. epidermidis* through disruption of bacterial membranes and confirming its anti-inflammatory efficacy in LPS-stimulated macrophages, BEO emerges as a multi-target therapeutic agent with both microbiological and immunomodulatory relevance. Network pharmacology further elucidated that the bioactivity of BEO is predominantly driven by borneol and modulated through key targets such as CYP19A1 and PPAR-α, primarily via metabolic signaling pathways. These insights not only deepen the mechanistic understanding of the action of BEO but also establish a scientific foundation for its potential application in treating inflammatory skin disorders. Future work may focus on in vivo efficacy and formulation development to translate these findings into use.

## Figures and Tables

**Table 1:** Minimum inhibitory concentrations (MIC) of BEO, NCB and previous reports of *Tanacetum argyrophyllum* var. *argyrophyllum* essential oil and *Salvia eremophila* essential oil together with common component analysis. (Unit: mg/mL).

	Gentamicin	BEO	NCB	*T. argyrophyllum* Essential Oil	*S. eremophila* Essential Oil
MIC against *S. epidermidis*	0.5	0.5	0.5	0.5	0.125
Main compounds (content %)					
Borneol		16.4	98.4	15	21.8
β-Caryophyllene		10.7	-	0.3	4.5
Camphor		10.6	0.8	26.6	-
Limonene		8.2	-	0.3	2.7
α-Pinene		7.5	-	2.4	18.8
Myrcene		6.2	-	-	0.6
Camphene		4.4	-	4.5	5.5
γ-Terpinene		3.7	-	0.5	0.6
Bicyclogermacrene		2.8	-	-	1.5
Terpinolene		1.6	-	0.5	0.3
β-Pinene		1.4	-	1.3	0.9
Spathulenol		0.9	-	0.9	1.2
Sabenene		0.9	-	0.3	-
Caryophyllene oxide		0.8	-	0.4	-
Linalool		0.5	-	-	1.5
α-Terpinene		0.4	-	0.2	0.3
Bornyl acetate		0.2	-	3.3	18.7
Globulol		0.1	-	-	2.9
Aromadendrene		0.1	-	-	1.7
References	Ebrahimabadi et al., 2010 [8]	Xiao et al., 2020 [1]		Polatoglu, 2010 [10]	Ebrahimabadi et al., 2010 [8]

BEO: *Cinnamomum camphora* chvar. *Borneol* essential oil; NCB: Natural crystalline borneol; “-”: not detected.

## Data Availability

The original contributions presented in this study are included in the article. Further inquiries can be directed to the corresponding author.

## Supplementary Materials

The following supporting information can be downloaded at: https://www.mdpi.com/article/10.3390/plants14121880/s1, Figure S1: Inflammation pharmacology network of the “compounds–targets–pathways” regulated by natural crystalline borneol; Figure S2: Bactericidal effects of BEO and NCB on *S. epidermidis* using CLSM and Live/Dead BacLight viability staining (SYTO9/PI); Figure S3: Inflammation pharmacology network of the “compounds–targets–pathways” regulated by *Tanacetum argyrophyllum var. argyrophyllum* essential oil; Figure S4: Inflammation pharmacology network of the “compounds–targets–pathways” regulated by *Salvia eremophila* essential oil; Figure S5: Images of *C. camphora* trees; Table S1: Chemical composition of BEO; Table S2: Components, targets and pathways of inflammation regulated by BEO; Table S3: Components, targets and pathways of inflammation regulated by NCB; Table S4: Components, targets and pathways of inflammation regulated by *Tanacetum argyrophyllum var. argyrophyllum* essential oil; Table S5: Components, targets and pathways of inflammation regulated by *Salvia eremophila* essential oil; Table S6: Network pharmacological analysis of inflammation with the number of targets and pathways regulated by BEO, NCB and previous reports of *Tanacetum argyrophyllum* var. *argyrophyllum* essential oil and *Salvia eremophila* essential oil.

## Abbreviations

The following abbreviations are used in this manuscript:

BEO	*Cinnamomum camphora* chvar. *Borneol* essential oil
NCB	Natural crystalline borneol
MIC	Minimum inhibitory concentration
LD	Linear dichroism
*μ*_max_	Maximum growth rate
SEM	Scanning electron microscopy
TEM	Transmission electron microscopy
LPS	Lipopolysaccharide
DMSO	Dimethyl sulfoxide
TNF-α	Tumor necrosis factor-α
(IL)-1β	Interleukin-1β
ELISA	Enzyme-linked immunosorbent assay
CFU	Colony Forming Units
CLSM	Confocal laser scanning microscopy
FBS	Fetal bovine serum
IC_50_	Half maximal inhibitory concentration
CYP	Cytochrome P450
SEM	Standard error of mean
PPAR-α	Peroxisome proliferator-activated receptor-α
CNR2	Cannabinoid receptor 2
TRP	Transient receptor potential cation channel V1
CTW	Component target weight
CPW	Component pathway weight
