# Supplementary material for: The Antibacterial and Anti-Inflammatory Potential of Cinnamomum camphora chvar. Borneol Essential Oil In Vitro
Source: Plants (Basel). 2025 Jun 19;14(12):1880. doi: 10.3390/plants14121880 (PMC12196741; doi:10.3390/plants14121880)
Supplement: Supplementary file 1 [file plants-14-01880-s001.zip › Fig.S1.pptx]

## Slide 1
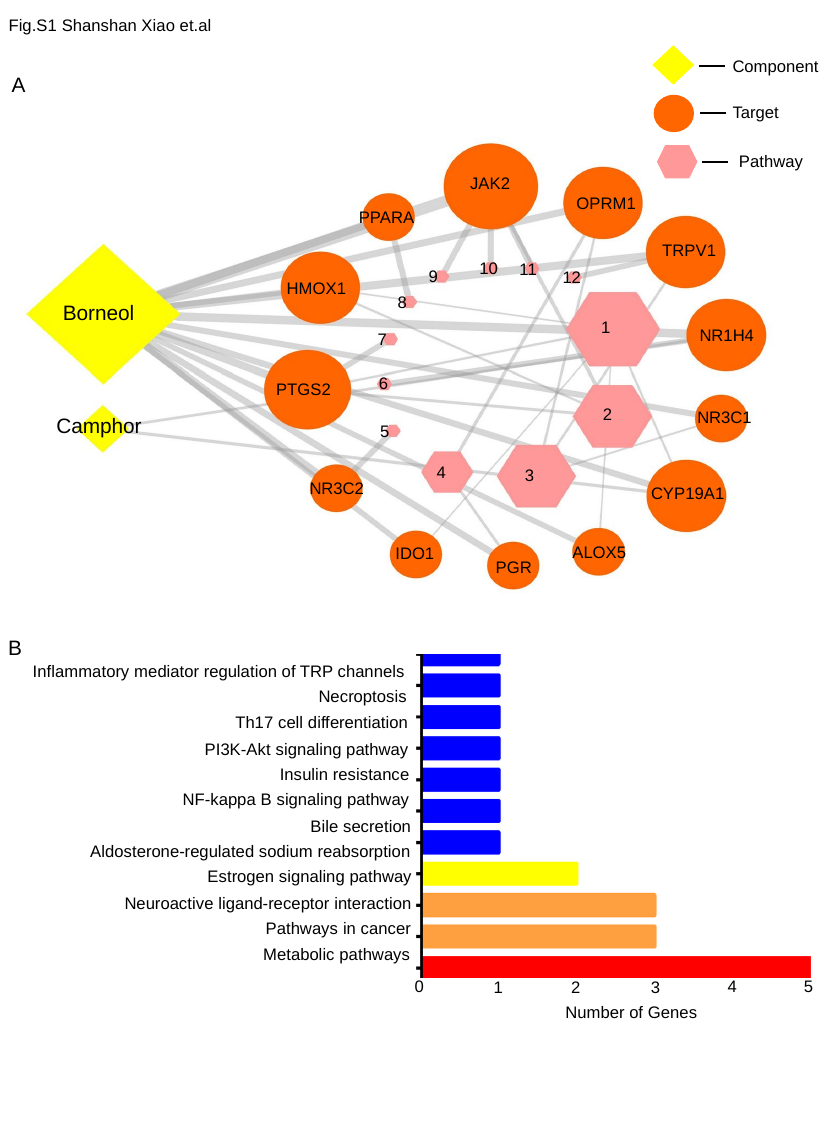

Fig.S1 Shanshan Xiao et.al
Component
A
Target
Pathway
JAK2
OPRM1
PPARA
TRPV1
10
11
9
12
HMOX1
8
Borneol
1
NR1H4
7
6
PTGS2
2
NR3C1
Camphor
5
4
3
NR3C2
CYP19A1
ALOX5
IDO1
PGR
B
Inflammatory mediator regulation of TRP channels
Necroptosis
Th17 cell differentiation
PI3K-Akt signaling pathway
Insulin resistance
NF-kappa B signaling pathway
Bile secretion
Aldosterone-regulated sodium reabsorption
Estrogen signaling pathway
Neuroactive ligand-receptor interaction
Pathways in cancer
Metabolic pathways
5
0
4
2
3
1
Number of Genes
