# Supplementary material for: The Antibacterial and Anti-Inflammatory Potential of Cinnamomum camphora chvar. Borneol Essential Oil In Vitro
Source: Plants (Basel). 2025 Jun 19;14(12):1880. doi: 10.3390/plants14121880 (PMC12196741; doi:10.3390/plants14121880)
Supplement: Supplementary file 1 [file plants-14-01880-s001.zip › Fig.S3 (2).pptx]

## Slide 1
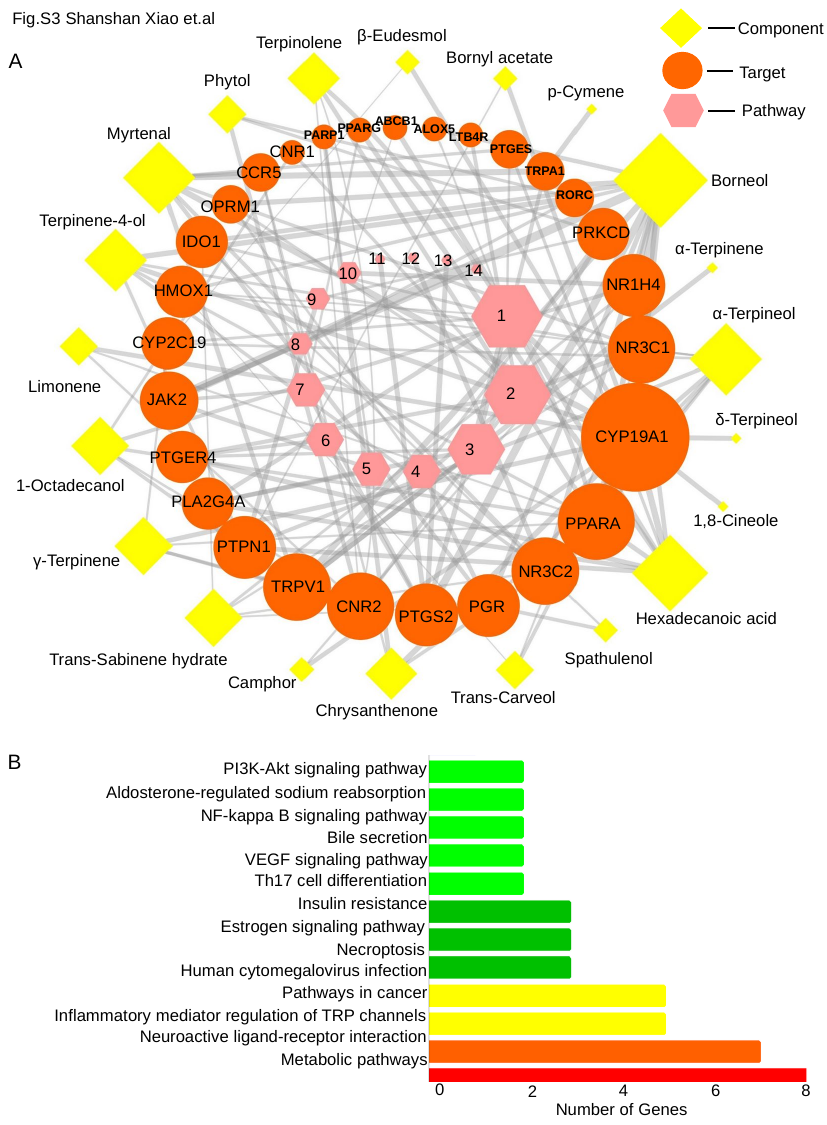

Fig.S3 Shanshan Xiao et.al
Component
β-Eudesmol
Terpinolene
Bornyl acetate
A
Target
Phytol
p-Cymene
Pathway
ABCB1
PPARG
ALOX5
Myrtenal
PARP1
LTB4R
CNR1
PTGES
CCR5
TRPA1
Borneol
RORC
OPRM1
Terpinene-4-ol
PRKCD
IDO1
α-Terpinene
12
11
13
14
10
NR1H4
HMOX1
9
α-Terpineol
1
CYP2C19
8
NR3C1
Limonene
7
2
JAK2
δ-Terpineol
CYP19A1
6
3
PTGER4
5
4
1-Octadecanol
PLA2G4A
1,8-Cineole
PPARA
PTPN1
γ-Terpinene
NR3C2
TRPV1
CNR2
PGR
PTGS2
Hexadecanoic acid
Spathulenol
Trans-Sabinene hydrate
Camphor
Trans-Carveol
Chrysanthenone
B
PI3K-Akt signaling pathway
Aldosterone-regulated sodium reabsorption
NF-kappa B signaling pathway
Bile secretion
VEGF signaling pathway
Th17 cell differentiation
Insulin resistance
Estrogen signaling pathway
Necroptosis
Human cytomegalovirus infection
Pathways in cancer
Inflammatory mediator regulation of TRP channels
Neuroactive ligand-receptor interaction
Metabolic pathways
0
4
6
8
2
Number of Genes
