# Supplementary material for: The Antibacterial and Anti-Inflammatory Potential of Cinnamomum camphora chvar. Borneol Essential Oil In Vitro
Source: Plants (Basel). 2025 Jun 19;14(12):1880. doi: 10.3390/plants14121880 (PMC12196741; doi:10.3390/plants14121880)
Supplement: Supplementary file 1 [file plants-14-01880-s001.zip › Fig.S4 (2).pptx]

## Slide 1
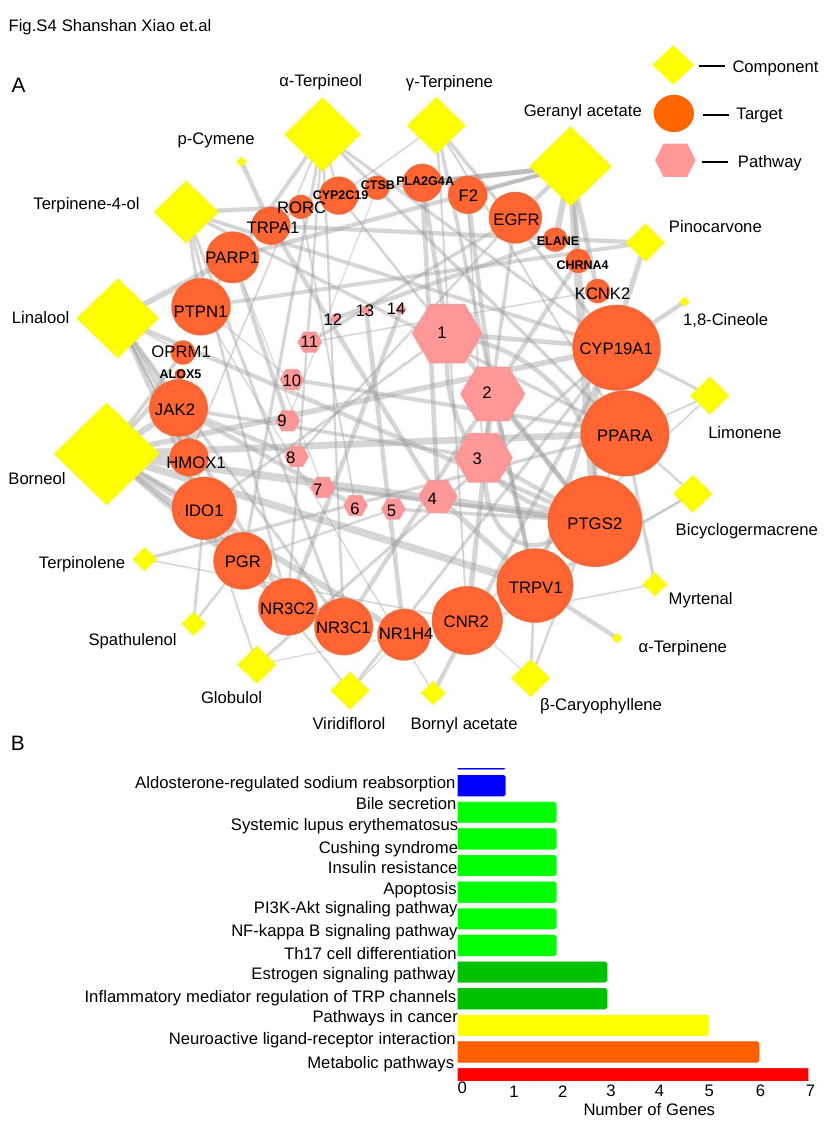

Fig.S4 Shanshan Xiao et.al
Component
α-Terpineol
γ-Terpinene
A
Geranyl acetate
Target
p-Cymene
Pathway
PLA2G4A
CTSB
F2
CYP2C19
Terpinene-4-ol
RORC
EGFR
Pinocarvone
TRPA1
ELANE
PARP1
CHRNA4
KCNK2
14
13
PTPN1
Linalool
1,8-Cineole
12
1
11
CYP19A1
OPRM1
ALOX5
10
2
JAK2
9
Limonene
PPARA
8
3
HMOX1
Borneol
7
4
6
IDO1
5
PTGS2
Bicyclogermacrene
PGR
Terpinolene
TRPV1
Myrtenal
NR3C2
CNR2
NR3C1
NR1H4
Spathulenol
α-Terpinene
Globulol
β-Caryophyllene
Viridiflorol
Bornyl acetate
B
Aldosterone-regulated sodium reabsorption
Bile secretion
Systemic lupus erythematosus
Cushing syndrome
Insulin resistance
Apoptosis
PI3K-Akt signaling pathway
NF-kappa B signaling pathway
Th17 cell differentiation
Estrogen signaling pathway
Inflammatory mediator regulation of TRP channels
Pathways in cancer
Neuroactive ligand-receptor interaction
Metabolic pathways
0
3
4
5
6
7
1
2
Number of Genes
