# Supplementary material for: The Antibacterial and Anti-Inflammatory Potential of Cinnamomum camphora chvar. Borneol Essential Oil In Vitro
Source: Plants (Basel). 2025 Jun 19;14(12):1880. doi: 10.3390/plants14121880 (PMC12196741; doi:10.3390/plants14121880)
Supplement: Supplementary file 1 [file plants-14-01880-s001.zip › Table S1.pdf]

Table S1. Chemical composition of BEO.

Classification	Compounds	Retention time (min)	Retention Index		Relative content (%)
			Measured	Documented[1]	
Monoterpenes	α-Pinene	4.85	931	934	7.45
	α-Thujene	4.9	941	945	0.99
	Camphene	5.56	948	948	4.43
	β-Pinene	6.3	981	981	3.57
	Sabenene	6.52	970	972	0.9
	3-Carene	7.04	1005	1009	0.43
	Myrcene	7.34	1003	1003	6.23
	γ-Terpinene	7.38	1014	1014	3.66
	α-Terpinene	7.6	1017	1020	0.38
	Limonene	8	1027	1030	8.23
	Trans-ocimene	8.58	1027	1027	0.1
	Trans-beta-ocimene	8.89	1050	1050	0.5
	Cymene	9.25	1032	1032	3.16
	Terpinolene	9.49	1078	1078	1.61
Oxygenated monoterpenes	Trans sabinene hydrate	12.56	1053	1053	0.06
	α-Ylangene	12.98	1375	1373	0.09
	Linalool	13.8	1084	1084	0.46
	Bornyl acetate	14.45	1285	1285	0.17
	β-Fenchyl alcohol	16.17	1569	1568	2.21
	Borneol	16.28	1698	1698	16.41
	Neryl alcohol	17.54	1785	1785	0.2
	Geraniol	18.16	1247	1247	0.15
Sesquiterpenes	α-Cubebene	12.53	1352	1352	0.14
	β-Elemene	14.63	1387	1387	0.41
	β-Caryophyllene	14.82	1389	1389	10.71
	Aromandendrene	14.95	1440	1440	0.11
	Humulene	15.89	1179	1179	3.97
	Germacrene	16.47	1802	1802	2.05
	α-Selinene	16.61	1664	1664	0.7
	γ-Elemene	18.1	1433	1435	0.63
Oxygenated sesquiterpenes	Camphor	13.53	1148	1146	10.6
	δ-Cadinene	17.06	1530	1524	0.24
	Caryophyllene oxide	20.13	1565	1560	0.8
	Humulene epoxide II	20.82	1391	1400	0.25
	Globulol	21.19	1569	1573	0.12
	Himbaccol	21.3	1604	1604	0.1
	Spathulenol	21.74	1617	1608	0.88

Classification	Compounds	Retention time (min)	Retention Index		Relative content (%)
			Measured	Documented[1]	
Others	α-Cadinol	22.99	1653	1650	0.12
	Juniper camphor	22.25	1691	1691	0.22
	α-Fenchene	5.41	953	953	0.2
	Bicyclogermacrene	16.78	1495	1493	2.83
	Methyl eugenol	20.27	1589	1589	0.26

BEO: *Cinnamomum camphora* chvar. *Borneol* essential oil; [1]: Retention index documented in the National Institute of Standards and Technology (NIST) WebBook Database (https://webbook.nist.gov/chemistry/)
