# Supplementary material for: The Antibacterial and Anti-Inflammatory Potential of Cinnamomum camphora chvar. Borneol Essential Oil In Vitro
Source: Plants (Basel). 2025 Jun 19;14(12):1880. doi: 10.3390/plants14121880 (PMC12196741; doi:10.3390/plants14121880)
Supplement: Supplementary file 1 [file plants-14-01880-s001.zip › Table S2.pdf]

Table S2. Components, targets and pathways of inflammation regulated by BEO.

Components	Targets		Pathways	
	Number	Name	Number	Name
Borneol	13	NR1H4, NR3C1, CYP19A1, NR3C2, PGR, IDO1, PTGS2, ALOX5, HMOX1, PPARA, JAK2, OPRM1, TRPV1	12	Insulin resistance, Inflammatory mediator regulation of TRP channels, Estrogen signaling pathway, Metabolic pathways, Bile secretion, Neuroactive ligand-receptor interaction, Pathways in cancer, PI3K-Akt signaling pathway Necroptosis, Th17 cell differentiation, Aldosterone-regulated sodium reabsorption, NF-kappa B signaling pathway
Linalool	9	NR3C2, NR3C1, PGR, HMOX1, IDO1, PTGS2, OPRM1, PARP1, JAK2	9	Estrogen signaling pathway, Metabolic pathways, Neuroactive ligand-receptor interaction, Pathways in cancer, PI3K-Akt signaling pathway, Necroptosis, Th17 cell differentiation, Aldosterone-regulated sodium reabsorption, NF-kappa B signaling pathway
α-Terpinene	8	CYP19A1, PTPN1, CYP2C19, NR3C2, NR3C1, PPARA, HMOX1, PGR	6	Insulin resistance, Estrogen signaling pathway, Neuroactive ligand-receptor interaction, Metabolic pathways, Pathways in cancer, Aldosterone-regulated sodium reabsorption
Methyl eugenol	7	CYP19A1, HMOX1, NOS3, BDKRB2, PARP1, JAK2, MIF	10	Metabolic pathways, Pathways in cancer, PI3K-Akt signaling pathway, Necroptosis, Th17 cell differentiation, NF-kappa B signaling pathway, Insulin resistance, Estrogen signaling pathway, Neuroactive ligand-receptor interaction, Inflammatory mediator regulation of TRP channels
α-Cadinol	6	CYP19A1, PTPN1, CYP2C19, RORC, PPARG, PPARA	4	Metabolic pathways, Insulin resistance, Th17 cell differentiation, Pathways in cancer
α-Selinene	6	CYP19A1, PPARA, CNR2, CYP2C19, PTPN1, TRPV1	4	Neuroactive ligand-receptor interaction, Inflammatory mediator regulation of TRP channels, Metabolic pathways, Insulin resistance
α-Ylangene	6	PPARA, CNR2, CYP19A1, CYP2C19, PTPN1, TRPV1	4	Neuroactive ligand-receptor interaction, Inflammatory mediator regulation of TRP channels, Metabolic pathways, Insulin resistance

Components	Targets		Pathways	
	Number	Name	Number	Name
3-Carene	5	PPARA, CNR2, TRPV1, CYP19A1, CYP2C19	4	Insulin resistance, Neuroactive ligand-receptor interaction, Inflammatory mediator regulation of TRP channels, Metabolic pathways
α-Thujene	5	PPARA, CNR2, TRPV1, CYP19A1, CYP2C19	4	Insulin resistance, Neuroactive ligand-receptor interaction, Inflammatory mediator regulation of TRP channels, Metabolic pathways
γ-Terpinene	5	PPARA, CNR2, TRPV1, CYP19A1, CYP2C19	4	Insulin resistance, Neuroactive ligand-receptor interaction, Inflammatory mediator regulation of TRP channels, Metabolic pathways
α-Cubebene	4	PPARA, CNR2, CYP19A1, CYP2C19	3	Insulin resistance, Neuroactive ligand-receptor interaction, Metabolic pathways
Bicyclogermacrene	3	PPARA, CNR2, TRPV1	3	Insulin resistance, Neuroactive ligand-receptor interaction, Inflammatory mediator regulation of TRP channels
Limonene	3	PPARA, CNR2, CYP19A1	3	Metabolic pathways, Insulin resistance, Neuroactive ligand-receptor interaction
Geraniol	3	PTGS2, PGR, JAK2	7	Estrogen signaling pathway, PI3K-Akt signaling pathway, Necroptosis, Th17 cell differentiation, Metabolic pathways, Pathways in cancer, NF-kappa B signaling pathway
Globulol	3	IDO1, CYP19A1, NR1H4	2	Metabolic pathways, Bile secretion
Himbaccol	3	IDO1, CYP19A1, NR1H4	2	Metabolic pathways, Bile secretion
Humulene	3	PPARA, CNR2, TRPV1	3	Insulin resistance, Neuroactive ligand-receptor interaction, Inflammatory mediator regulation of TRP channels
Juniper camphor	3	CYP19A1, PTPN1, CYP2C19	2	Metabolic pathways, Insulin resistance
β-Caryophyllene	3	PPARA, CNR2, TRPV1	3	Insulin resistance, Neuroactive ligand-receptor interaction, Inflammatory mediator regulation of TRP channels
γ-Elemene	3	PPARA, CNR2, CXCR3	3	Insulin resistance, Neuroactive ligand-receptor interaction, Viral protein interaction with cytokine and cytokine receptor

Components	Targets		Pathways	
	Number	Name	Number	Name
Bornyl acetate	2	PTPN1, CYP19A1	2	Metabolic pathways, Insulin resistance
Camphor	2	CYP19A1, NR1H4	2	Metabolic pathways, Bile secretion
Spathulenol	2	IDO1, PGR	2	Estrogen signaling pathway, Metabolic pathways
Terpinolene	2	PPARA, CNR2	2	Insulin resistance, Neuroactive ligand-receptor interaction
Trans-Ocimene	2	PPARA, CNR2	2	Insulin resistance, Neuroactive ligand-receptor interaction
Myrcene	2	PPARA, CNR2	2	Insulin resistance, Neuroactive ligand-receptor interaction
Cymene	1	TRPA1	1	Inflammatory mediator regulation of TRP channels
Germacrene	1	NR3C1	1	Neuroactive ligand-receptor interaction
Humulene epoxide II	1	NLRP3	1	Necroptosis
β-Elemene	1	CXCR3	1	Viral protein interaction with cytokine and cytokine receptor

BEO: *Cinnamomum camphora* chvar. *Borneol* essential oil
