# Supplementary material for: The Antibacterial and Anti-Inflammatory Potential of Cinnamomum camphora chvar. Borneol Essential Oil In Vitro
Source: Plants (Basel). 2025 Jun 19;14(12):1880. doi: 10.3390/plants14121880 (PMC12196741; doi:10.3390/plants14121880)
Supplement: Supplementary file 1 [file plants-14-01880-s001.zip › Table S3.pdf]

Table S3. Components, targets and pathways of inflammation regulated by NCB.

**Compounds**	**Target**		**Pathway**	
	**Number**	**Name**	**Number**	**Name**
Borneol	13	NR1H4, NR3C1, CYP19A1, NR3C2, PGR, IDO1, PTGS2, ALOX5, HMOX1, PPARA, JAK2, OPRM1, TRPV1	12	Insulin resistanceInflammatory mediator regulation of TRP channels, Estrogen signaling pathway, Metabolic pathways, Bile secretion, Neuroactive ligand-receptor interaction, Pathways in cancer, PI3K-Akt signaling pathway, Necroptosis, Th17 cell differentiation, Aldosterone-regulated sodium reabsorption, NF-kappa B signaling pathway
Camphor	2	CYP19A1, NR1H4	2	Metabolic pathways, Bile secretion

NCB: natural crystalline borneol
