# Supplementary material for: The Antibacterial and Anti-Inflammatory Potential of Cinnamomum camphora chvar. Borneol Essential Oil In Vitro
Source: Plants (Basel). 2025 Jun 19;14(12):1880. doi: 10.3390/plants14121880 (PMC12196741; doi:10.3390/plants14121880)
Supplement: Supplementary file 1 [file plants-14-01880-s001.zip › Table S4.pdf]

Table S4. Components, targets and pathways of inflammation regulated by *Tanacetum argyrophyllum var. argyrophyllum* essential oil

**Compounds**	**Target**		**Pathway**	
	**Number**	**Name**	**Number**	**Name**
Borneol	13	NR1H4, NR3C1, CYP19A1, NR3C2, PGR, IDO1, PTGS2, ALOX5, HMOX1, PPARA, JAK2, OPRM1, TRPV1	13	Bile secretion, Neuroactive ligand-receptor interaction, Metabolic pathways, Aldosterone-regulated sodium reabsorption - Homo sapiens (human), Estrogen signaling pathway, NF-kappa B signaling pathway, Human cytomegalovirus infection, Pathways in cancer, VEGF signaling pathway, Necroptosis, PI3K-Akt signaling pathway - Homo sapiens (human), Th17 cell differentiation, Inflammatory mediator regulation of TRP channels
Myrtenal	11	NR3C2, NR3C1, PGR, CYP19A1, TRPV1, CCR5, PTGES, TRPA1, PTPN1, CYP19A1, LTB4R	7	Neuroactive ligand-receptor interaction, Metabolic pathways, Aldosterone-regulated sodium reabsorption - Homo sapiens (human), Estrogen signaling pathway, Inflammatory mediator regulation of TRP channels, Insulin resistance, Human cytomegalovirus infection
Hexadecanoic acid	9	PPARA, NR1H4, CYP19A1, PTPN1, PPARG, PTGER4, ABCB1, PTGES, PLA2G4A	8	Bile secretion, Metabolic pathways, Insulin resistance, Pathways in cancer, Neuroactive ligand-receptor interaction, Human cytomegalovirus infection, Inflammatory mediator regulation of TRP channels, Necroptosis
α-Terpineol	8	CYP19A1, PTPN1, CYP2C19, NR3C2, NR3C1, PPARA, HMOX1, PGR	5	Neuroactive ligand-receptor interaction, Metabolic pathways, Aldosterone-regulated sodium reabsorption - Homo sapiens (human), Estrogen signaling pathway, Insulin resistance
Terpinene-4-ol	6	CYP19A1, RORC, PTPN1, PPARA, NR3C1, NR3C2	5	Neuroactive ligand-receptor interaction, Metabolic pathways, Aldosterone-regulated sodium reabsorption - Homo sapiens (human), Insulin resistance, Th17 cell differentiation
γ-Terpinene	5	PPARA, CNR2, TRPV1, CYP19A1, CYP2C19	3	Metabolic pathways, Neuroactive ligand-receptor interaction, Inflammatory mediator regulation of TRP channels
1-Octadecanol	5	NR1H4, CNR1, TRPV1, CNR2, PPARA	3	Bile secretion, Neuroactive ligand-receptor interaction, Inflammatory mediator regulation of TRP channels

Compounds	Target		Pathway	
	Number	Name	Number	Name
Trans-sabinene hydrate	5	NR3C1, NR1H4, NR3C2, PGR, IDO1	5	Bile secretion, Neuroactive ligand-receptor interaction, Aldosterone-regulated sodium reabsorption - Homo sapiens (human), Estrogen signaling pathway, Metabolic pathways
Chrysanthenone	4	CYP19A1, CCR5, NR3C2, PARP1	4	Metabolic pathways, Aldosterone-regulated sodium reabsorption - Homo sapiens (human), Human cytomegalovirus infection, Necroptosis
Limonene	3	PPARA, CNR2, CYP19A1	2	Metabolic pathways, Neuroactive ligand-receptor interaction
Phytol	3	PRKCD, CNR2, RORC	5	Neuroactive ligand-receptor interaction, Estrogen signaling pathway, Inflammatory mediator regulation of TRP channels, Insulin resistance, Th17 cell differentiation
Trans-carveol	3	CYP19A1, HMOX1, NR3C1	2	Neuroactive ligand-receptor interaction, Metabolic pathways
Bornyl acetate	2	PTPN1, CYP19A1	2	Metabolic pathways, Insulin resistance
Camphor	2	CYP19A1, NR1H4	2	Bile secretion, Metabolic pathways
Spathulenol	2	IDO1, PGR	2	Estrogen signaling pathway, Metabolic pathways
Terpinolene	2	PPARA, CNR2	1	Neuroactive ligand-receptor interaction
β-Eudesmol	2	CYP19A1, CYP2C19	1	Metabolic pathways
1,8-Cineole	1	CYP19A1	1	Metabolic pathways
p-Cymene	1	TRPA1	1	Inflammatory mediator regulation of TRP channels
α-Terpinene	1	TRPV1	1	Inflammatory mediator regulation of TRP channels
δ-Terpineol	1	CYP19A1	1	Metabolic pathways
