# Supplementary material for: The Antibacterial and Anti-Inflammatory Potential of Cinnamomum camphora chvar. Borneol Essential Oil In Vitro
Source: Plants (Basel). 2025 Jun 19;14(12):1880. doi: 10.3390/plants14121880 (PMC12196741; doi:10.3390/plants14121880)
Supplement: Supplementary file 1 [file plants-14-01880-s001.zip › Table S5.pdf]

Table S5. Components, targets and pathways of inflammation regulated by *Salvia eremophila* essential oil.

Compounds	Target		Pathways	
	Number	Name	Number	Name
Borneol	13	NR1H4, NR3C1, CYP19A1, NR3C2, PGR, IDO1, PTGS2, ALOX5, HMOX1, PPARA, JAK2, OPRM1, TRPV1	11	Metabolic pathways, Insulin resistance, Inflammatory mediator regulation of TRP channels, Neuroactive ligand-receptor interaction, Bile secretion, Aldosterone-regulated sodium reabsorption, Estrogen signaling pathway, NF-kappa B signaling pathway, Pathways in cancer, Th17 cell differentiation, PI3K-Akt signaling pathway
Linalool	9	NR3C2, NR3C1, PGR, HMOX1, IDO1, PTGS2, OPRM1, PARP1, JAK2	9	Neuroactive ligand-receptor interaction, Aldosterone-regulated sodium reabsorption, Estrogen signaling pathway, Metabolic pathways, NF-kappa B signaling pathway, Pathways in cancer, Apoptosis, Th17 cell differentiation, PI3K-Akt signaling pathway
Geranyl acetate	9	CTSB, PLA2G6, F2, PTGS2, EGFR, ELANE, PARP1, CHRNA4, KCNK2	9	Metabolic pathways, NF-kappa B signaling pathway, Pathways in cancer, Estrogen signaling pathway, Neuroactive ligand-receptor interaction, Apoptosis, Inflammatory mediator regulation of TRP channels, Systemic lupus erythematosus, Cushing syndrome
α-Terpineol	8	CYP19A1, PTPN1, CYP2C19, NR3C2, NR3C1, PPARA, HMOX1, PGR	6	Metabolic pathways, Neuroactive ligand-receptor interaction, Aldosterone-regulated sodium reabsorption, Estrogen signaling pathway, Insulin resistance, Pathways in cancer
Terpinene-4-ol	6	CYP19A1, RORC, PTPN1, PPARA, NR3C1, NR3C2	5	Metabolic pathways, Insulin resistance, Neuroactive ligand-receptor interaction, Aldosterone-regulated sodium reabsorption, Th17 cell differentiation, Insulin resistance
γ-Terpinene	5	PPARA, CNR2, TRPV1, CYP19A1, CYP2C19	4	Metabolic pathways, Insulin resistance, Neuroactive ligand-receptor interaction, Inflammatory mediator regulation of TRP channels
Bicyclogermacrene	3	PPARA, CNR2, TRPV1	3	Insulin resistance, Neuroactive ligand-receptor interaction, Inflammatory mediator regulation of TRP channels

Compounds	Target		Pathways	
	Number	Name	Number	Name
Limonene	5	PPARA, CNR2, CYP19A1	3	Metabolic pathways, Insulin resistance, Neuroactive ligand-receptor interaction
Globulol	3	IDO1, CYP19A1NR1H4	2	Metabolic pathways, Bile secretion
β-Caryophyllene	3	PPARA, CNR2, TRPV1	3	Insulin resistance, Neuroactive ligand-receptor interaction, Inflammatory mediator regulation of TRP channels
Pinocarvone	3	PTPN1, TRPV1, TRPA1	3	Neuroactive ligand-receptor interaction, Inflammatory mediator regulation of TRP channels, Insulin resistance
Viridiflorol	3	IDO1, CYP19A1, NR1H4	2	Metabolic pathways, Bile secretion
Bornyl acetate	2	PTPN1, CYP19A1	2	Metabolic pathways, Insulin resistance
Spathulenol	2	IDO1, PGR	2	Estrogen signaling pathway, Metabolic pathways
Terpinolene	2	PPARA, CNR2	2	Insulin resistance, Neuroactive ligand-receptor interaction
Myrcene	2	PPARA, CNR2	2	Insulin resistance, Neuroactive ligand-receptor interaction
α-Terpinene	1	TRPV1	2	Inflammatory mediator regulation of TRP channels, Neuroactive ligand-receptor interaction
1,8-Cineole	1	CYP19A1	1	Metabolic pathways
p-Cymene	1	TRPA1	1	Inflammatory mediator regulation of TRP channels
