# Supplementary material for: The Antibacterial and Anti-Inflammatory Potential of Cinnamomum camphora chvar. Borneol Essential Oil In Vitro
Source: Plants (Basel). 2025 Jun 19;14(12):1880. doi: 10.3390/plants14121880 (PMC12196741; doi:10.3390/plants14121880)
Supplement: Supplementary file 1 [file plants-14-01880-s001.zip › Table S6.pdf]

Table S6. Network pharmacological analysis of inflammation with the number of targets and pathways regulated by BEO, NCB and previous reports of *Tanacetum argyrophyllum var. argyrophyllum* essential oil and *Salvia eremophila* essential oil.

Components	BEO			NCB			*T. argyrophyllum* essential oil			*S. eremophila* essential oil		
	C (%)	T (%)	P (%)	C (%)	T (%)	P (%)	C (%)	T (%)	P (%)	C (%)	T (%)	P (%)
Borneol	16.4	55.83	55.1	98.4	99.88	99.86	15	66.17	67.9	21.8	68.28	65.59
β-Caryophyllene	10.7	8.53	8.99	-	-	-	-	-	-	4.5	3.25	3.69
Limonene	8.2	6.54	6.89	-	-	-	0.3	0.31	0.21	2.7	1.95	2.22
Camphor	10.6	5.53	5.94	0.8	0.12	0.14	26.6	17.68	18.52	-	-	-
γ-Terpinene	3.7	4.88	4.14	-	-	-	0.5	0.85	0.52	0.6	0.72	0.66
Myrcene	6.2	3.23	3.47	-	-	-	-	-	-	0.6	0.28	0.33
Humulene	4	3.19	3.36	-	-	-	-	-	-		-	-
Bicyclogermacrene	2.8	2.23	2.35	-	-	-	-	-	-	1.5	1.08	1.23
α-Thujene	0.99	1.31	1.11	-	-	-	-	-	-	-	-	-
Linalool	0.5	1.18	1.26	-	-	-	-	-	-	1.5	3.25	3.69
α-Selinene	0.7	1.09	0.78	-	-	-	-	-	-	-	-	-
Cymene	3.2	0.88	0.9	-	-	-	-	-	-	-	-	-
α-Terpinene	0.4	0.83	0.67	-	-	-	0.2	0.07	0.07	0.3	0.07	0.08
Terpinolene	1.6	0.83	0.9	-	-	-	0.2	0.13	0.07	0.3	0.14	0.16
Germacrene	2.1	0.58	0.59	-	-	-	-	-	-	-	-	-
3-Carene	0.43	0.57	0.48	-	-	-	-	-	-	-	-	-
Methyl eugenol	0.3	0.55	0.84	-	-	-	-	-	-	-	-	-
γ-Elemene	0.6	0.48	0.5	-	-	-	-	-	-	-	-	-
Spathulenol	0.9	0.47	0.5	-	-	-	0.9	0.6	0.63	1.2	0.57	0.66
α-Cadinol	0.12	0.19	0.13	-	-	-	-	-	-	-	-	-
Juniper camphor	0.2	0.16	0.11	-	-	-	-	-	-	-	-	-
α-Cubebene	0.14	0.15	0.12	-	-	-	-	-	-	-	-	-
α-Ylangene	0.09	0.14	0.1	-	-	-		-	-	-	-	-

Components	BEO			NCB			*T. argyrophyllum* essential oil			*S. eremophila* essential oil		
	C (%)	T (%)	P (%)	C (%)	T (%)	P (%)	C (%)	T (%)	P (%)	C (%)	T (%)	P (%)
Geraniol	0.15	0.12	0.29	-	-	-	-	-	-	-	-	-
β-Elemene	0.4	0.11	0.11	-	-	-	-	-	-	-	-	-
Bornyl acetate	0.2	0.1	0.11	-	-	-	3.3	2.19	2.3	18.7	8.87	10.23
Globulol	0.1	0.08	0.06	-	-	-	-	-	-	2.9	2.09	1.59
Himbaccol	0.1	0.08	0.06	-	-	-	-	-	--	-	-	-
Humulene epoxide	0.3	0.08	0.08	-	-	-	-	-	-	-	-	-
Trans-Ocimene	0.1	0.05	0.06	-	-	-	-	-	-	-	-	-
1,8-Cineole	-	-	-	-	-	-	1.3	0.43	0.45	3	0.74	1.64
p-Cymene	-	-	-	-	-	-	1.2	0.4	0.42	0.9	0.22	0.25
α-Terpineol	-	-	-	-	-	-	1.1	2.99	1.92	1	1.92	1.64
Terpinene-4-ol	-	-	-	-	-	-	1.9	3.85	3.31	1.8	2.6	2.46
Trans-sabinene	-	-	-	-	-	-	0.3	0.51	0.52	-	-	--
β-Eudesmol	-	-	-	-	-	-	0.6	0.4	0.21	-	-	-
Chrysanthenone		-	-	-	-	-	0.2	0.27	0.28	-	-	-
Trans-carveol	-	-	-	-	-	-	0.2	0.21	0.14	-	-	-
1-Octadecanol	-	-	-	-	-	-	0.1	0.17	0.1	-	-	-
Phytol	-	-	-	-	-	-	0.1	0.1	0.17	-	-	-
δ-Terpineol	-	-	-	-	-	-	0.3	0.1	0.1	-	-	-
Hexadecanoic acid	-	-	-	-	-	-	0.6	1.83	1.67	-	-	-
Myrtenal	-	-	-	-	-	-	0.2	0.75	0.49	-	-	-
Viridiflorol	-	-	-	-	-	-	-	-	-	2.3	1.66	1.26
Pinocarvone	-	-	-	-	-	-	-	-	-	0.2	0.14	0.16
Geranyl acetate	-	-	-	-	-	-	-	-	-	1	2.16	2.46
References	Xiao et al.,2020						Polatoglu, 2010			Ebrahimabadi et al., 2010		

BEO: *Cinnamomum camphora* chvar. *Borneol* essential oil; NCB: Natural crystalline borneol; "-": not detected; "C%": represents content; "T%": represents target contribution weight; "P%": represents pathway contribution weight
