# Supplementary figures and images for: The Antibacterial and Anti-Inflammatory Potential of Cinnamomum camphora chvar. Borneol Essential Oil In Vitro
Source: Plants (Basel). 2025 Jun 19;14(12):1880. doi: 10.3390/plants14121880 (PMC12196741; doi:10.3390/plants14121880)

## Slide 1
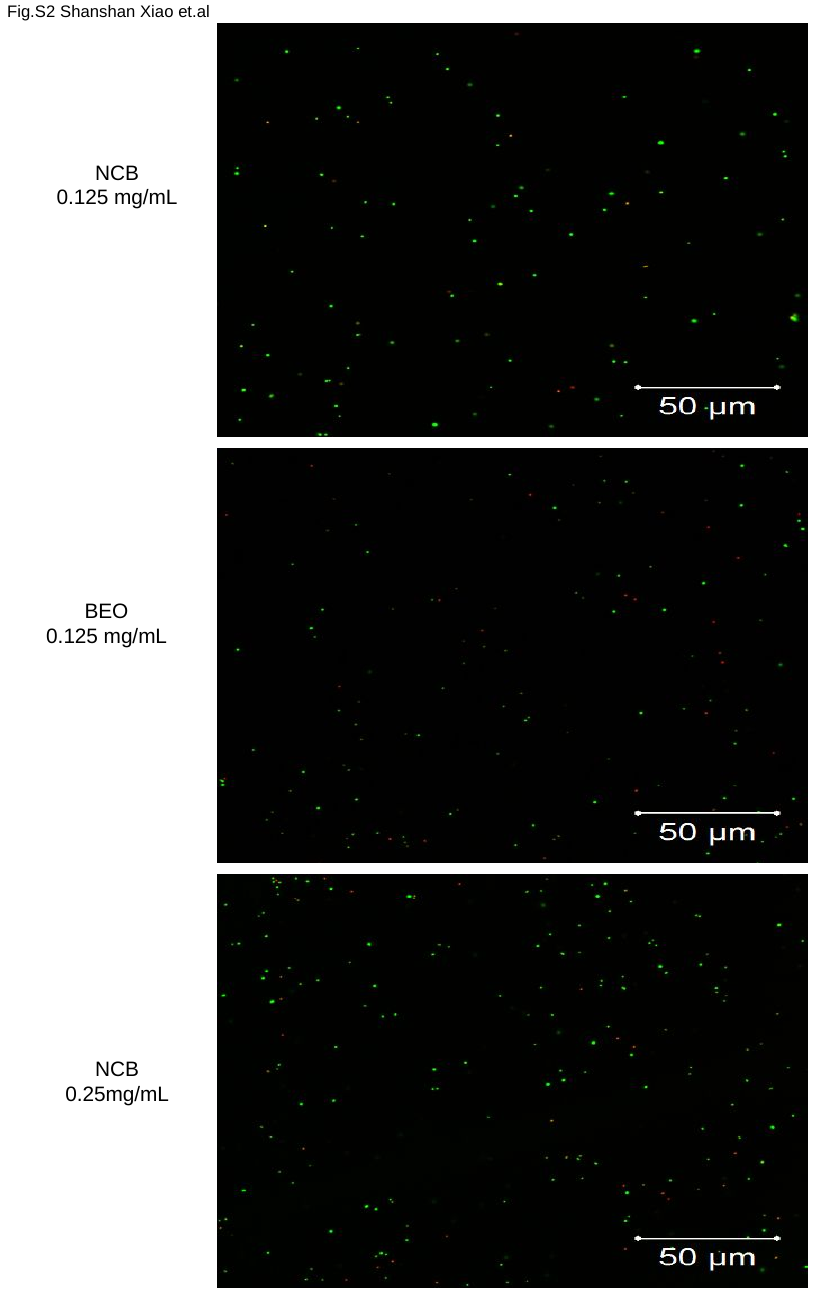

Fig.S2 Shanshan Xiao et.al
NCB
0.125 mg/mL
BEO
0.125 mg/mL
NCB
0.25mg/mL

Supplement: Supplementary file 1 [file plants-14-01880-s001.zip › Fig.S2 (2).pptx]

## Slide 1
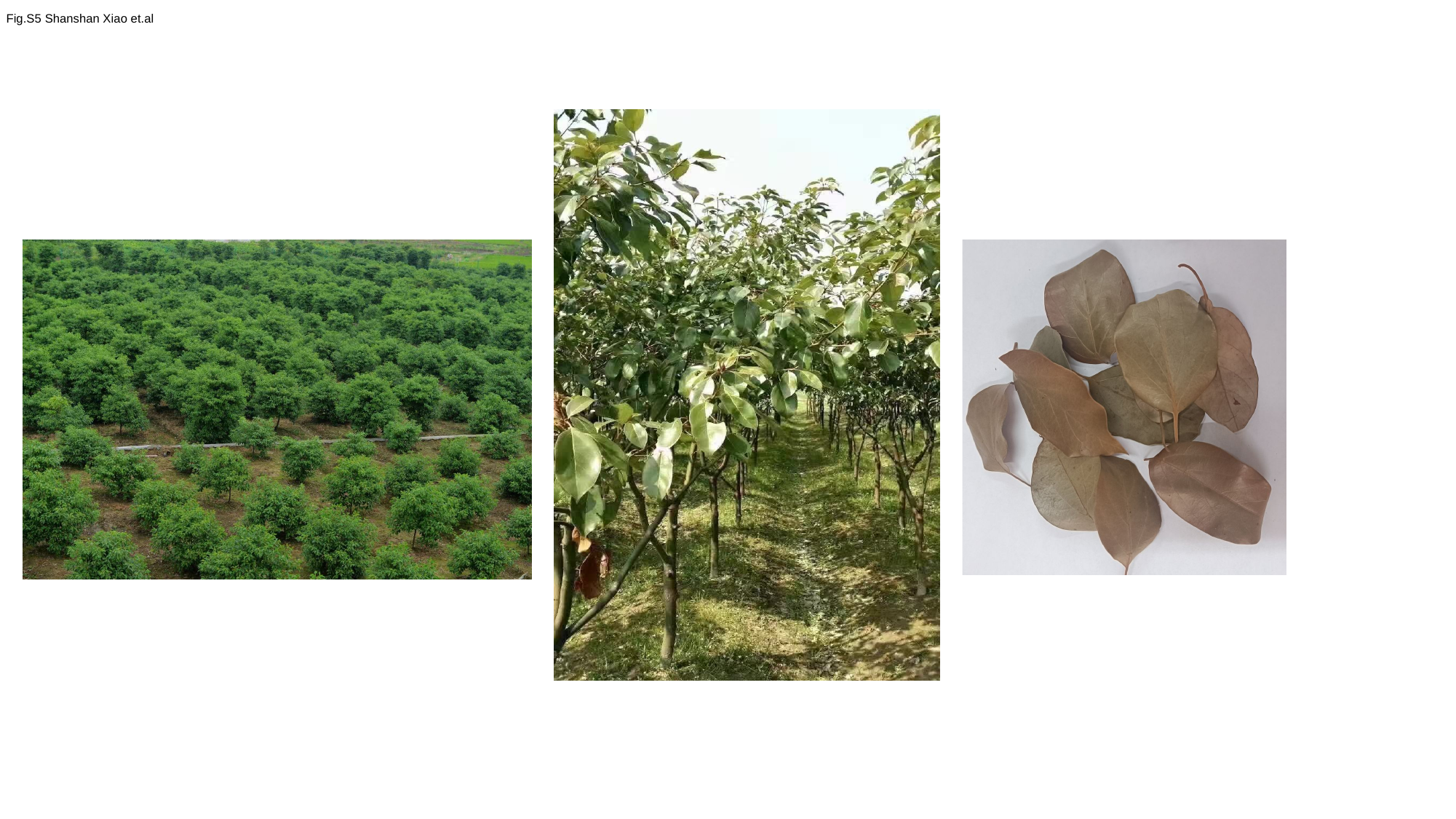

Fig.S5 Shanshan Xiao et.al

Supplement: Supplementary file 1 [file plants-14-01880-s001.zip › Fig.S5.pptx]
